# Recent Advances in SAW Sensors for Detection of Cancer Biomarkers

**DOI:** 10.3390/bios15020088

**Published:** 2025-02-05

**Authors:** Manuel Aleixandre, Mari Carmen Horrillo

**Affiliations:** 1 Institute of Integrated Research (IIR), Institute of Science Tokyo, Suzukakedai Campus, Yokohama 226-0026, Japan; 2 SENSAVAN, Instituto de Tecnologías Físicas y de la Información (ITEFI), Consejo Superior de Investigaciones Científicas (CSIC), 28006 Madrid, Spain

**Keywords:** surface acoustic wave (SAW), cancer, biosensors, cancer biomarkers, sensors, VOCs

## Abstract

Surface acoustic wave (SAW) sensor technology is a promising approach to diagnosing cancer through the detection of cancer biomarkers due to its high sensitivity, potential label-free operation, and fast response times, and, fundamentally, because it is a non-invasive technique in comparison with the current traditional diagnostic techniques for cancer. This review focuses on this application, and for this purpose, the recent literature on cancer biomarkers detected by this advanced technology has been compiled, including that on volatile organic compounds (VOCs) from exhaled breath and larger biomolecules such as proteins, DNA, and microRNAs in body fluids, which demonstrates its great versatility. The conventional techniques for cancer biomarker detection in biofluids, such as ELISA, PCR, SPR, and UV absorbance, exhibit limitations including high costs, slow response times, a reduced sensitivity, the need for specialized instrumentation, and the requirement for highly trained personnel. Different SAW sensor configurations are discussed with attention paid to their specific properties, wave propagation modes, and suitability for different environments. Detailed studies are reviewed, highlighting biomarkers for lung, colorectal, prostate, breast, and ovarian cancer diagnostics, as well as the detection of circulating tumor cells and cancerous cell growth. This review identifies current challenges, including optimizing sensitivity, addressing environmental interferences, and the need for clinical validation. Finally, future research directions are proposed, emphasizing the use of VOC biomarkers and the integration of SAW technology into hybrid systems and microfluidic platforms to enable the creation of scalable, non-invasive diagnostic tools for the detection of cancer in early stages, and, in this way, to minimize the morbidity and mortality associated with this disease.

## 1. Introduction

Cancer is a leading cause of mortality worldwide according to the WHO [1], and its early detection is critical for improving survival rates [1]. Advances in diagnostic technologies aim to identify the disease at its earliest stages [2,3,4]. Biomarkers are being investigated that are associated with specific biological or chemical changes associated with cancer [5,6,7,8,9]. Surface acoustic wave (SAW) [10] sensors [11], operating through the propagation of acoustic waves on piezoelectric materials, have shown great potential for this purpose. Their high sensitivity to changes in mass and viscosity properties at the sensor surface makes them ideal candidates for detecting cancer biomarkers [12,13,14].

SAW sensors are versatile and can detect a wide range of biomarkers, from volatile organic compounds (VOCs) in exhaled breath to larger biomolecules such as proteins and DNA found in body fluids. They offer several advantages such as label-free detection, real-time operation, and compatibility with miniaturized and portable systems, making them promising for use in non-invasive early diagnosis. In contrast, the traditional techniques widely used for cancer detection, such as biopsy and imaging techniques, cytological analysis, and endoscopic procedures, are invasive and, in general, show limited sensitivity, low specificity, and a high cost, and do not detect cancer in its early stages [15].

Moreover, and more recently, biomarkers in bodily fluids (mostly in blood) are being used as indicators of cancer due to their minimal invasiveness. The most commonly used biomarkers are PSA, CEA, and CA-125/MUC16, while exosomes, microRNA, and circulating tumor cells are gaining recognition as promising new biomarkers [8].

Regarding biomarkers in gaseous environments, VOCs stand out as the most promising due to their capacity to indicate metabolic changes linked to tumor development. This non-invasive method shows potential for early cancer diagnosis. Cancer-associated VOCs include alkanes, aldehydes, ketones, and aromatic hydrocarbons, which are linked to processes such as oxidative stress, lipid peroxidation, and disrupted cellular metabolism [16].

The detection of these cancer biomarkers by traditional techniques such as gas chromatography-mass spectrometry for VOCs, or ELISA and PCR for biomarkers in bodily fluids, has similar limitations, including long analysis times and high operational costs.

These limitations, summarized in Table 1, highlight the need for more accurate, non-invasive, and efficient diagnostic techniques, such as the development of novel biosensors. In this paper we only refer to the use of SAW biosensors to detect cancer, presenting a detailed review of all relevant studies on this niche topic identified in the existing literature. Other alternative biosensors include other acoustic ones, as well as electrochemical, resistive, colorimetric, and optical sensors, and these have been applied to detect many types of cancer, such as breast, lung, colon, prostate, ovarian, esophageal, and liver cancer. The biomarker types that they detect include proteins, VOCs, nucleic acids, cytokines, circulating tumor cells (CTCs), circulating tumor DNA (ctDNA), and other small molecules [6,7].

Numerous reviews exist on the development and application of SAW sensors [11,13], primarily focusing on their general principles, device configurations, and material properties. These reviews provide an extensive foundation for understanding the underlying mechanisms of SAW sensors. In the biosensing domain, reviews often highlight the sensitivity of SAW sensors in detecting biomolecules like proteins, DNA, and cells [14,17,18], but these works typically do not focus on the cancer-specific domain.

This paper aims to address this gap by analyzing the potential of SAW technology in detecting cancer biomarkers. This paper discusses the fundamental principles of SAW technology, detailing the types of devices, piezoelectric materials, and guiding layers that optimize its performance. In addition, recent advancements in the functionalization of sensor surfaces to improve their specificity and sensitivity are highlighted. Through a review of key studies, the current challenges and opportunities for using SAW sensors in cancer diagnostics are analyzed, emphasizing their potential for integration into clinical diagnostic platforms.

## 2. SAW Sensor Devices and Their Key Features

SAW devices operate based on the propagation of acoustic waves along the surface of a piezoelectric material, such as quartz or lithium niobate, when an oscillating electric field is applied. This technology has versatile applications; one of the most common uses is in SAW filters in telecommunications. SAW filters leverage carefully designed inter-digitated transducers (IDTs) [19] to selectively transmit or block specific frequencies, making them essential components in wireless communication systems where precise signal filtering is required. SAW sensor devices are specifically designed to exploit the surface sensitivity of SAW technology. By applying a chemically selective layer on the device surface, SAW sensors can be made to detect target analytes, such as gases or biological markers, by capturing them on this coating. When these analytes bind to the surface, they cause detectable shifts in the wave properties, such as its frequency or amplitude, based on changes in the sensor’s surface mass, viscosity, or electrical characteristics. These shifts enable SAW sensors to identify and quantify specific analytes, making them ideal for application in environmental monitoring and medical diagnostics, where their high sensitivity to trace compounds is a significant advantage.

Acoustic sensors like quartz crystal microbalance (QCM), film bulk acoustic resonator (FBAR), and bulk acoustic wave (BAW) sensors operate on different principles to measure mass or mechanical changes. QCM sensors use the piezoelectric effect in quartz crystals to detect very small mass variations by monitoring frequency shifts, making them useful for biosensing and material analysis. FBAR sensors are thin-film devices that generate bulk acoustic waves in a piezoelectric layer, often featuring a “floating” structure to improve their sensitivity, which makes them suitable for compact applications like RF filtering and use as portable biosensors. BAW sensors produce acoustic waves through the bulk of a material, allowing them to work well in conditions where surface contamination could affect the performance of other sensors, such as in telecommunications and medical diagnostics. These types of sensors, however, are not reviewed in this paper, as the focus is on SAW sensors [20,21].

### 2.1. Types of SAW Devices

**Figure 1:**
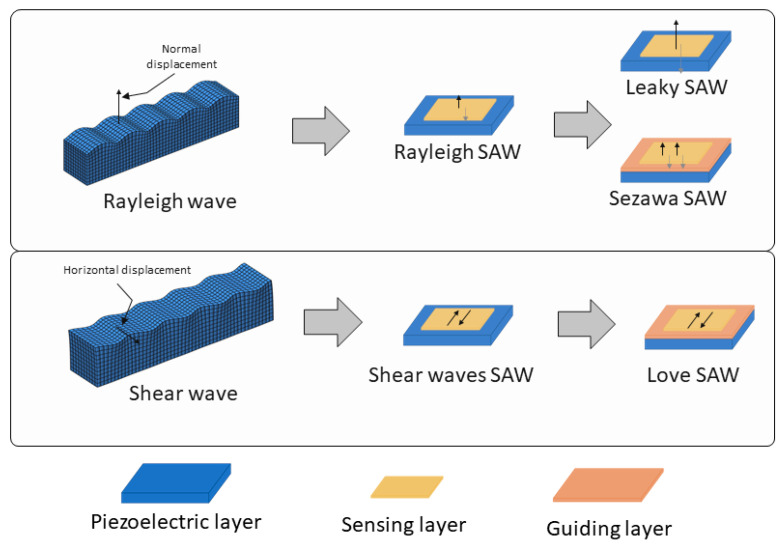
Scheme of the types of SAW devices according to the surface acoustic wave propagation modes, (**top**) Rayleigh type and (**bottom**) shear mode. In the figure, the different configurations, including the disposition of the guiding layer (depicted as a pink layer) in the Sezawa and Love SAW sensors can be seen. Thin black arrows represent wave displacements.

The biomarkers for cancer cover a wide range, from small molecules, such as VOCs [22,23], to whole cells [24,25], as well as proteins [26], RNA [27], and DNA [28] molecules found in both air and liquid samples. Considering this diversity, the sensory characteristics required for SAW biosensing must be versatile and precisely tuned to detect these varied targets. Regarding the types of surface acoustic waves, several kinds of devices are employed in biosensing, each with distinct wave propagation modes that improve the sensitivity and selectivity for different biomarker types [29] (Figure 1).

Two main wave propagation modes are employed in SAW sensor devices. Rayleigh waves are the most traditional type [26,30], producing surface-confined waves with both vertical and horizontal displacement components, which makes them highly sensitive to changes in surface mass and viscosity. Due to their high sensitivity to surface loading, Rayleigh SAW devices experience increased signal losses when used in liquid environments, making them more suitable for sensing in air. Due to their careful design, selecting the appropriate cut, piezoelectric material, and IDT configuration that is parallel to the wave propagation, shear horizontal SAW (SH-SAW) devices generate waves that move horizontally parallel to the surface, which minimizes their sensitivity to liquid loading. This makes SH-SAW devices particularly well suited for biosensing in liquid environments [31,32,33], such as in blood or saliva analysis.

Other advanced SAW configurations can be considered subtypes of these two main wave modes. Among the devices which use Rayleigh-type waves, there are leaky-SAW devices, which are a variant of Rayleigh wave sensors where part of the wave energy “leaks” into the surroundings [25,34]. This is caused by strong piezoelectric materials cut in the appropriate angles, which causes the waves to leak energy into the medium as they travel. This characteristic makes them especially useful in liquid sensing despite the increased damping. Another Rayleigh-based subtype is Sezawa waves [35], which are higher-order Rayleigh waves that require a layered structure, which helps to confine the energy’s wave closer to the surface, typically with a thin guiding layer on the substrate, leading to enhanced sensitivity and higher operational frequencies but higher levels of noise, damping, and technical complexities.

**Figure 2:**
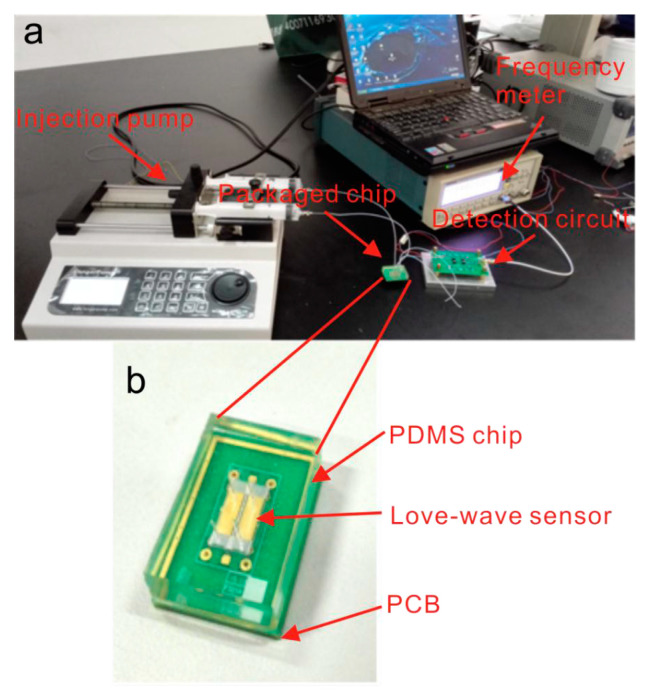
An image of a Love-SAW sensor from [38], illustrating a microfluidic Love-wave biosensing device. This device is built on a LiTaO₃ substrate with a SiO₂ waveguide layer, an aptamer-beacon probe for PSA detection, and PDMS microfluidic channels enabling real-time, label-free measurements. (**a**) Overall measurement system, (**b**) detail of the SAW sensor and the sensor chamber.

The Love-mode SAW devices, a subtype of the shear SAW devices, are by far the most common SAW type used in biomarker cancer detection [27,30,36,37]. Love-mode SAW devices guide the SH-SAW waves within a thin overlay layer on the piezoelectric substrate (as depicted in Figure 1). This structure traps wave energy near the surface, enhancing the sensor’s sensitivity to surface-bound analytes and making Love-mode devices particularly effective for detecting biomolecules in difficult liquid samples. Figure 2 shows an image of the measurement system used by this type of sensor to detect prostate-specific antigen (PSA).

The main characteristics of these types of SAW devices are presented in Table 2.

Lamb waves and flexural plate waves (FPWs) were not included in this review, as they are distinct from SAWs in that they propagate through the entire thickness of a thin membrane or plate rather than being confined to the surface. Additionally, while certain bulk acoustic waves (BAWs) have a leaky mode that allows a fraction of their energy to interact with the surface, they were excluded from this review because BAWs fundamentally differ from surface waves, as they propagate through the entire volume of the material rather than being limited to the surface.

### 2.2. Structural Characteristics of the SAW Devices

Regarding the piezoelectric material, quartz, lithium niobate, lithium tantalate, and zinc oxide are the most commonly used piezoelectric materials in SAW devices due to their complementary properties (Table 2). Quartz has exceptional thermal stability and cost-effectiveness, making it ideal when temperature stability is crucial. Lithium niobate has a strong piezoelectric coupling property, so it has a high sensitivity. Lithium tantalate displays a balance between thermal stability and moderate coupling, making its use versatile in sensor applications that require both stability and sensitivity. Finally, zinc oxide (ZnO) adds further versatility due to its strong piezoelectric response and ability to be deposited as a thin film. This makes ZnO especially valuable in wearable sensors where flexibility of the SAW is essential.

Also, wave-guiding layers play a key role in SAW device characteristics. Non-piezoelectric guiding layers, such as silicon dioxide (SiO₂) or polymethyl methacrylate (PMMA), are commonly used to confine wave energy close to the surface of the device. Their ability to do this depends on their wave propagation properties such as their attenuation and wave velocity. Non-piezoelectric layers are also cost-effective and easier to deposit, making them practical for use in general-purpose SAW applications. On the other hand, piezoelectric guiding layers, such as zinc oxide (ZnO) and aluminum nitride (AlN) [49], enable coupling between electric fields and acoustic waves, which allows for additional tuning. However, they are more difficult to deposit as they need to be suited to the crystallization to have piezoelectric properties.

The IDT configurations are essential in SAW device design, as modifications to the finger spacing, orientation, and number of electrodes directly influence wave characteristics such as the frequency, amplitude, and propagation mode. Narrower finger spacing enables higher-frequency wave generation, thereby increasing the device’s sensitivity. Custom IDT configurations are also critical for generating SH waves, where both the piezoelectric substrate and proper alignment of the IDT in the correct direction are crucial for effective shear wave generation. In Sezawa and leaky SAW devices, the IDT configuration controls the wave frequency, where accuracy is essential for proper wave excitation.

### 2.3. Configurations: Delay Lines versus Resonator

**Figure 3:**
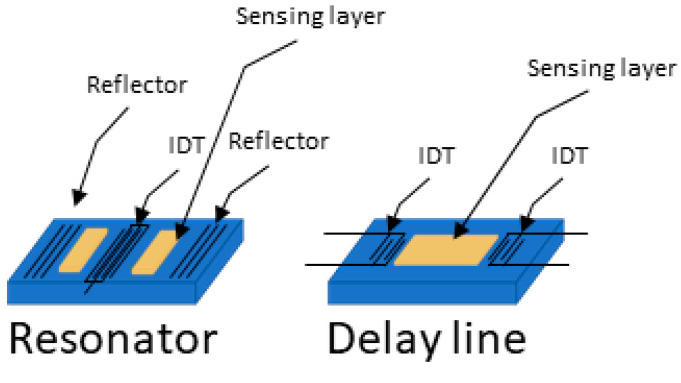
Resonator and delay line SAW device configurations. (**Left**) resonator configuration where the wave generated is a steady wave, (**right**) where the wave travels from one IDT to the other.

In SAW sensors, there are two main configurations: delay lines and resonators, shown in Figure 3. Each has specific advantages and limitations which can make them better suited for detecting different types of cancer biomarkers depending on their size and the surrounding environment (gas or liquid).

Delay lines consist of two IDTs, one to send the acoustic wave and one to receive it, with a set path in between. This design provides a broad frequency response, which is useful for detecting various changes, such as shifts in mass, viscosity, or temperature. Delay lines are simpler to manufacture and work well for continuous monitoring, especially in gas environments. In gases, where there is minimal damping of the wave, delay lines are very sensitive to small molecules, such as VOCs, that can be linked to cancers (like in breath analysis for lung cancer). Delay lines can also work in liquid environments, where they are better suited for detecting larger biomarkers, such as proteins or even whole cells, because their broad frequency range captures the large shifts caused by these bigger molecules. However, delay lines have a lower frequency stability, which may cause signal drift over time, particularly in liquids where there is more damping.

Resonators, on the other hand, use reflective gratings to trap waves between two IDTs, creating a stable standing wave at a specific resonant frequency. Reflectors are typically used to induce resonance in SAW devices. In some designs, negative reflectors, created, for example, by alternating open and shorted strips to shift the wave phase by 180 degrees, can be employed to better confine acoustic energy within the resonator [50]. Resonators are very sensitive to small frequency shifts, making them ideal for detecting tiny changes in mass, which is useful for sensing small cancer biomarkers like metabolites or proteins in liquid samples. Resonators are also less affected by liquid damping, which allows them to make stable, precise measurements even in viscous liquids like blood. However, resonators have a narrower frequency response and are more complex to fabricate, which makes them less adaptable for real-time, general sensing or for detecting large biomarkers like cells.

In summary, delay lines are better for general-purpose sensing, especially in gases or for larger biomarkers like cells and proteins. Resonators are best for applications that need high stability and sensitivity, especially in liquids, and are ideal for detecting small molecules or proteins.

### 2.4. Sensing Layers in SAW Sensors

The sensitive layer in SAW biosensors is fundamental for improving their performance since it interacts with the specific analytes. This layer is sometimes functionalized with biomolecules like antibodies [36,44,51,52,53], aptamers [25,43], or DNA probes [27,54] that have a high affinity for some biomarkers of interest. Other sensitive materials are also employed to detect VOCs in air or cells such as polymers [22,42], molecular-imprinted polymers [23,37,55,56,57], and carbon structures [30,58], and the devices are even used uncoated [59,60] when selectivity can be obtained by other methods. When the target analyte binds or is adsorbed to the sensitive layer, it causes a detectable change in the mass or mechanical properties.

For functionalization, the more straightforward approach is the direct immobilization of sensing molecules, such as antibodies or proteins, directly onto the surface of the SAW device [28,44,45,50]. This is usually achieved through methods like physical adsorption and covalent bonding techniques.

Physical adsorption relies on non-covalent interactions, such as electrostatic forces, hydrophobic interactions, and Van der Waals forces, to hold the biomolecules onto the sensor surface. This method is relatively straightforward but may result in weak and reversible attachments. As a particular example, glucose oxidase immobilized via adsorption on platinum-multiwalled carbon nanotube nanocomposites exhibited a good sensitivity of 113.13 μA mM^−1^ cm^−2^ but suffered from reduced stability over extended use [61].

On the other hand, covalent bonding techniques involve forming strong chemical bonds between functional groups on the sensor surface and the reactive groups on the capture molecules. Common strategies include using cross-linking agents or activating the surface with chemical treatments to introduce reactive groups. For example, in [60], the authors immobilized tyrosinase on a glassy carbon electrode modified with electrodeposited gold nanoparticles to achieve a high sensitivity, a high stability, a rapid response, and reproducibility in detecting phenolic compounds, achieving a lifetime stability of 18 days. Covalent attachment provides a more stable and durable linkage, which is advantageous for long-term sensor applications.

However, direct immobilization has certain limitations. The random orientation of immobilized biomolecules can affect their binding sites’ accessibility, potentially reducing the sensor’s sensitivity. Additionally, the activity of the immobilized molecules may be compromised if their active sites are obstructed or if they undergo conformational changes upon attachment. The non-specific binding of other substances present in the sample can also interfere with the sensor’s selectivity, leading to false signals or increased background noise [62].

To overcome the challenges associated with direct immobilization, a different approach is commonly used. Intermediate layer modifications are introduced as a mediating layer between the SAW surface and the captured molecules. These create an environment that enhances the specific capture of analytes while minimizing non-specific interactions, although they can lower the sensitivity. For example, incorporating a gold layer [39,41] onto the sensor surface acts as an interface between the device substrate and the capture molecules, facilitating efficient biomolecule immobilization. Gold allows for the formation of self-assembled monolayers (SAMs) through creating strong thiol–gold bonds, enabling the covalent and oriented attachment of biomolecules like antibodies or DNA probes. This controlled immobilization preserves the biological activity of the capture molecules and enhances the sensitivity and selectivity of the sensor. Additionally, the gold layer provides chemical stability and reduces non-specific adsorption, leading to improved sensor performance in applications such as medical diagnostics. Molecules like dextran or GPTES [27,28] provide binding points on the surface that can reduce non-specific adsorption. They also offer functional groups for the covalent attachment of biomolecules in a controlled orientation, preserving their biological activity. Polymers and hydrogels [36,42], being composed of interconnected networks, create three-dimensional matrixes that can immobilize a high density of capture molecules. Their porous nature facilitates the diffusion of analytes to the binding sites while maintaining the structural integrity of the immobilized biomolecules. This results in an enhanced steric stability and preserves the functionality of the capture molecules.

Recent studies have demonstrated the synergistic use of advanced materials such as nanomaterials, including graphene and carbon nanotubes, to address these limitations. These materials enhance the surface area, the device’s biocompatibility, and the signal amplification, improving the immobilization efficiency and biosensor sensitivity. Advances in combining immobilization methods, such as combining adsorption with covalent bonding or nanotechnology, are fundamental for achieving superior sensor performance, and recent reviews explore this important aspect in more detail [62,63].

**Figure 4:**
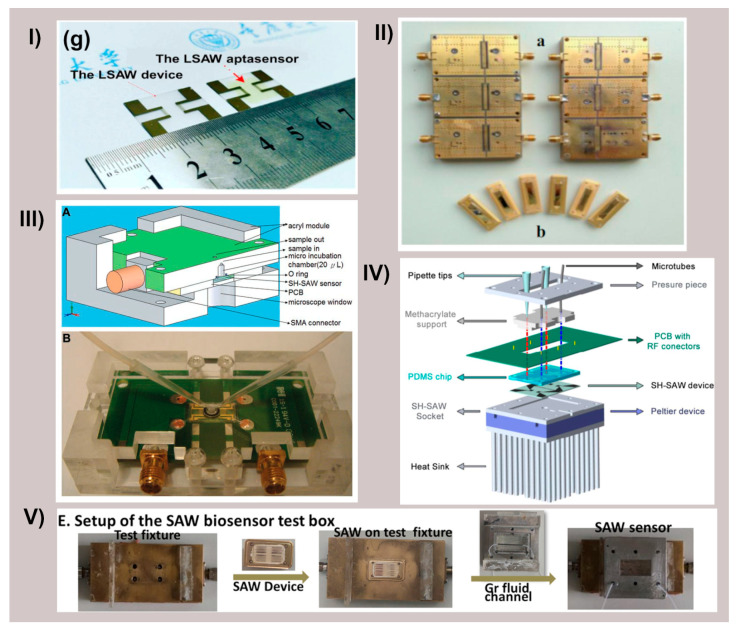
Different SAW devices and their fluidic configurations. (**I**) Panel (g) of [43] representing the LSAW chip and the LSAW aptasensor developed. Reprinted with permission from Ref. [43]. Copyright 2022 Elsevier. (**II**) Schematic diagram of 2 × 3 model of LSAW aptasensor array; (a) 2 × 3 model of sensor array, (b) photograph of a LSAW atpasensor from [25]. Reprinted with permission from Ref. [25]. Copyright 2014 Elsevier. (**III**) Schematic illustration of shear horizontal surface acoustic wave (SH-SAW) sensors and the detection system. (A) Cross-section of polymethylmethacrylate mold with SH-SAW sensor. (B) SH-SAW sensors to detect antigen-specific cells from [53]. Reprinted with permission from Ref. [53]. Copyright 2013 Elsevier. (**IV**) Liquid cell diagram showing the different components from [64]. (**V**) Panel E. from [30] assembly of the SAW immunosensor test box. Reprinted with permission from Ref. [30]. Copyright 2022 Elsevier.

Figure 4 shows several examples of SAW-based biosensor designs and setups that have been utilized for cancer biomarker detection, demonstrating the diversity of approaches in the field.

## 3. Biomarkers

Cancer biomarkers are molecules, genes, proteins, or other substances found in the body that can indicate the presence of, progression of, or response to cancer treatments. They have become increasingly significant in recent years due to their role in the diagnosis, prognosis, monitoring, and personalized treatment of cancer. A recent review of the main cancer biomarkers can be found in [8]. In general, biomarkers can be categorized based on their clinical applications and molecular characteristics. From a clinical perspective, diagnostic biomarkers are used to identify the presence of cancer, such as prostate-specific antigen (PSA) for prostate cancer. Prognostic biomarkers provide insights into the likely disease progression or risk of recurrence, exemplified by HER2/neu in breast cancer. Predictive biomarkers assess the likelihood of a patient’s response to specific therapies, such as EGFR mutations in non-small cell lung cancer, to assist in targeted treatments. Monitoring biomarkers can be done to track treatment responses or detect disease recurrence, with CA-125 being a notable example in ovarian cancer.

From a molecular standpoint, biomarkers include a range of types. The use of protein-based biomarkers, such as tumor antigens and tumor-related proteins, can be exemplified by the use of alpha-fetoprotein (AFP) for treating liver cancer. The use of genetic biomarkers involves detecting specific gene mutations or alterations, such as BRCA1 and BRCA2 mutations in breast and ovarian cancer. RNA-based biomarkers, including microRNAs, can indicate abnormal gene expression associated with cancer, such as microRNAs detected in liquid biopsies for lung cancer. VOCs, detected non-invasively in breath, may also indicate the presence of cancer, such as lung cancer. Metabolite biomarkers reflect tumor-related metabolic activity, as seen with 2-hydroxyglutarate in IDH-mutant tumors.

Additionally, biomarkers can be classified based on their origin. Circulating biomarkers, such as circulating tumor cells (CTCs) and tumor-derived free DNA, are detectable in blood or other bodily fluids. Tissue biomarkers, on the other hand, are identified in biopsy samples, with Ki-67 serving as a marker for cell proliferation in tumor tissues. This multifaceted classification highlights the diverse utility of biomarkers in cancer diagnosis, prognosis, and treatment monitoring.

**Figure 5:**
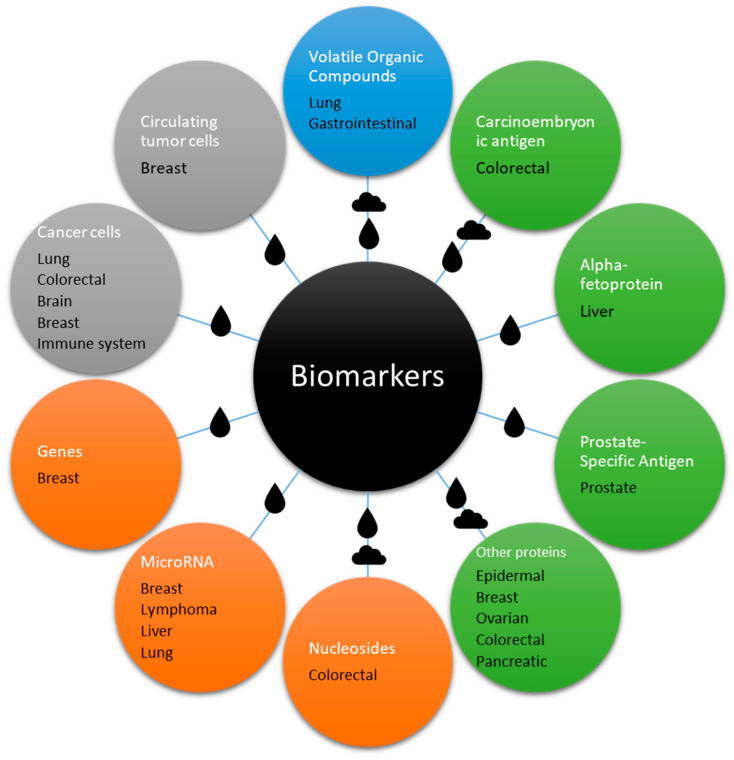
Scheme of the biomarkers detailed in this review in relation with the different types of cancer that they are associated with. The drop and cloud symbols represent the medium (liquid or gas) used to detect them, which in some cases is both. The color represents the general category: blue for small molecules, green for proteins, orange for genetic material, and grey for cells.

This review provides a comprehensive compilation, mostly of recent studies, focusing on cancer biomarkers detected using various types of SAW biosensors, as shown in Figure 5.

### 3.1. VOCs

This review highlights the potential of VOCs as biomarkers for early, non-invasive cancer detection. For over five decades, research has focused on VOCs emitted by the human body, with early work by Linus Pauling in 1971 identifying breath as containing around 250 VOCs. By 1999, studies by Phillips and colleagues expanded this number to over 3400 compounds. These VOCs, produced through metabolic processes, are carried to the lungs via the bloodstream and exhaled. As such, changes in breath composition can be linked to diseases, including cancer. Works on detecting lung cancer by detecting VOCs with SAW biosensors are predominant in the literature, due surely to the fact that it is often asymptomatic in its early stages, with symptoms like coughing, chest pain, and weight loss being commonly overlooked. As a result, 85% of cases are diagnosed at advanced stages, leading to a low five-year survival rate of around 10–15%. Despite advancements in diagnostic methods like CT scans and biopsies, late-stage detection limits treatment options. Besides, lung cancer produces 1.6 million deaths annually, surpassing the total number of deaths due to colon, prostate, and breast cancers. Detecting VOCs in breath with gas sensors, such as SAW biosensors, is one of the most interesting strategies for diagnosing this cancer at the beginning of the disease and in a non-invasive mode [65,66,67].

Also, gastrointestinal cancer accounts for 22.2% of global cancer-related deaths, with histological biopsy under endoscopy being the primary diagnostic method of this cancer. However, endoscopy has limitations, including its cost, invasiveness, and low diagnostic rates for early-stage cancer due to its nonspecific symptoms. Non-invasive, cost-effective diagnostic methods are urgently needed. Common alternatives like fecal occult blood tests, serum biomarkers (e.g., CEA, CA199), and gastrointestinal barium angiography also have drawbacks, including high false positive/negative rates, low accuracy, and procedural challenges. The need for reliable, non-invasive biomarkers is critical, with VOCs emerging as promising candidates due to their link to oxidative reactions in cancer cells, which spread through the blood to the respiratory system [68].

Gas sensors have shown significant promise in detecting lung cancer through the analysis of VOCs in exhaled breath. Arrays of MOS sensors and advanced nanosensors are commonly used for lung cancer detection due to their high sensitivity, robustness, and low cost. They detect VOCs like acetone, toluene, benzene, and formaldehyde in exhaled breath, which are associated with metabolic changes in lung cancer cells. These portable systems are integrated with machine learning algorithms to achieve improved accuracy in lung cancer diagnosis and to provide a non-invasive, real-time diagnostic option [69].

SAW sensors demonstrate significant potential for the detection of VOC biomarkers associated with cancer, owing to their exceptional sensitivity and selectivity, rapid real-time response capabilities, low costs, and portability, as well as their ability to operate at room temperature and facilitate label-free detection. However, their application in this field remains relatively limited when compared to other technologies such as metal oxide semiconductor (MOS) sensors or gas chromatography-mass spectrometry (GC-MS) [67]. The lower use of SAW sensors may be attributed to challenges in optimizing their design for specific VOCs and the dominance of alternative, more established methods in cancer biomarker detection. This highlights the need for further research to develop SAW sensors, particularly on their potential integration into compact, point-of-care diagnostic devices for clinical applications.

The following studies use SAW sensor technology for the diagnosis of lung cancer through the detection of VOCs in breath samples. In [22], a Love-SAW sensor was developed that incorporates silver-modified polypyrrole (Ag/PPy) nanoparticles to detect VOCS at room temperature. The sensor, built on an ST-cut quartz substrate, detects acetone, ethanol, and toluene with detection limits of 3 ppb, 5 ppb, and 20 ppb, respectively. The Ag/PPy sensor showed higher sensitivity than a PPy-only sensor, likely due to silver’s catalytic effect. Its sensitivity values were 910 Hz/ppm for acetone, 742 Hz/ppm for ethanol, and 340 Hz/ppm for toluene, with fast response times. The sensor exhibited low cross-sensitivity to other gases and a moderate performance in humid conditions. The study suggests that Ag/PPy-modified Love-SAW sensors exhibit significant potential for the detection of VOCs at parts-per-billion (ppb) concentrations, making them highly suitable for breath analysis applications. Also, in [70], the same authors developed a Love-SAW sensor that operates at 160 MHz with a dual-layer structure, including an SiO₂ guiding layer and a sensitive polypyrrole (PPy) layer modified with gold nanoparticles (Au NPs). These sensors detect ammonia and ethylene, which are biomarkers for cancer. The addition of Au NPs significantly improved the sensor’s sensitivity, lowering its detection limits to 67 ppb for ammonia and 87 ppb for ethylene. While the sensors showed high sensitivity in dry air, their response stability decreased with humidity, suggesting the need for humidity control. The Au-modified PPy sensors offer enhanced selectivity and reproducibility, and are promising for breath analysis and the monitoring of low-concentration VOCs.

In [58], a filter-based SAW sensor operating at 433.92 MHz was developed for the detection of decane, a VOC associated with lung cancer. The sensor incorporates a thin oxidized graphene (GO) film as the sensing layer, applied to the SAW device with a thickness of 150–200 nm. This GO layer confers high sensitivity, achieving a detection limit of 0.2 ppm and exhibiting rapid response times. The device demonstrated stable performance with minimal variations in insertion loss. The non-conductive nature of the GO film mitigates electromechanical coupling effects, enhancing its efficacy in detecting non-polar VOCs such as decane. These characteristics suggest the sensor’s potential for early lung cancer diagnosis through breath analysis.

Other studies highlight the potential of integrating sensors with complementary technologies, such as chromatography and MOS sensors, which, as previously noted, are widely employed for the detection of such biomarkers. For instance, in [60], a SAW–gas chromatography system was developed for the early screening of lung cancer through the analysis of exhaled breath. This system combines a 500 MHz SAW sensor with a gas chromatography (GC) module, enabling the rapid and sensitive detection of lung cancer biomarkers, with a detection limit of 1 picogram. By analyzing breath samples from 19 lung cancer patients and 19 healthy controls, the system successfully identified key VOCs, such as alkanes and benzene derivatives, which distinguish between the two groups. This non-invasive diagnostic approach demonstrates high sensitivity and holds significant potential for clinical point-of-care applications. In [59], a hybrid electronic nose system (HENS) is presented, combining MOS sensors and SAW sensors for the detection of lung cancer biomarkers in breath samples. The system utilizes nine MOS sensors to detect low-molecular-weight VOCs, such as benzene and hexane, and uncoated SAW sensors for the detection of high-molecular-weight VOCs, such as tridecane. Therefore, the innovative combination of these two types of gas sensors offers greater sensitivity to a wider range of VOC species in breath compared to the use of just one type of sensor. The SAW sensors, operating at 250 MHz on ST-cut quartz substrates, provide improved stability and are uncoated to minimize environmental interference. With temperature control and a differential structure, the system achieves high sensitivity (93.62%) and selectivity (83.37%) using an artificial neural network (ANN) model, demonstrating its potential as a promising tool for non-invasive lung cancer screening.

For gastrointestinal cancer detection, in [23], a Love wave sensor with a thin film of a molecularly imprinted polymer (MIP) as sensitive layer was developed for the detection of ethanol and toluene, targeting colorectal cancer diagnostics. Built on an AT-cut quartz substrate with a SiO₂ guiding layer, the sensor operates with shear-horizontal waves at a 40 μm wavelength. The MIP layer, imprinted with adenosine monophosphate (AMP), enhances the sensor’s selectivity, providing up to four times the sensitivity of non-imprinted polymer (NIP) sensors and faster response times. The sensor exhibits high specificity, indicating its potential for colorectal cancer biomarker detection.

As stated, VOCs (acetone, ethanol, toluene, ammonia, ethylene, decane, alkanes, benzene derivatives, hexane, and tridecane) are critical non-invasive biomarkers for early cancer detection. SAW sensors offer significant potential for diagnosing cancers such as lung and gastrointestinal cancers, due to their high sensitivity, high selectivity, and real-time detection capabilities. Compared to MOS sensors and gas chromatography, SAW sensors provide advantages like portability and room-temperature operation, making them well-suited for point-of-care applications. Challenges such as sensitivity to humidity and the need for optimized VOC targeting require further research, including research that allows for advancements in sensor design, signal processing, and clinical validation.

### 3.2. Carcinoembryonic Antigen

Carcinoembryonic antigen (CEA) is a well-established biomarker primarily associated with colorectal cancer, although it may also be elevated in other malignancies such as pancreatic, gastric, and lung cancers. In colorectal cancer, CEA is most often used to monitor disease progression, its recurrence, and its response to treatment rather than for initial diagnosis, as not all patients with colorectal cancer exhibit elevated levels. Elevated CEA levels after surgery, for example, may indicate cancer recurrence, making it a valuable tool in follow-up care [71].

#### 3.2.1. CEA Detection in Exhaled Breath Condensate

Reference [45] presents a portable Love-wave sensor developed for the detection of carcinoembryonic antigen (CEA) in exhaled breath condensate (EBC) for early lung cancer diagnosis. The sensor employs a CEA-specific aptamer immobilized on a gold-coated silicon dioxide layer, operating at a frequency of 164 MHz. It demonstrates high sensitivity (0.577°/(ng/mL)) and a detection limit of 1.04 ng/mL, with stable performance for up to 10 days. The sensor exhibits high specificity for CEA, with minimal interference from other lung cancer biomarkers such as neuron-specific enolase (NSE) and squamous cell carcinoma antigen (SCC). This approach integrates aptamer technology with a dual-channel Love-wave sensor, enabling label-free, real-time detection. Another study [52] introduces a Love-SAW immunosensor constructed on an ST-cut quartz substrate with a 3 µm SiO₂ guiding layer, operating at 160 MHz. The sensor utilizes a gold staining amplification method to enhance its sensitivity and employs a sandwich immunoassay with gold nanoparticle (AuNP) conjugates. The sensor offers a detection range from 1 to 16 ng/mL and a detection limit of 1 ng/mL. It demonstrates high selectivity against interfering agents such as NSE and SCC, and its performance shows a high correlation (0.999) with clinical chemiluminescent immunoassays. This device provides a rapid, non-invasive tool for early lung cancer screening in high-risk populations. In [51], a point-of-care diagnostic system is developed using a Love-SAW immunosensor with immunogold staining to detect lung cancer biomarkers. The sensor, built on an ST-cut quartz substrate with a SiO₂ guiding layer, operates at 165 MHz with gold IDTs and utilizes a dual-SAW setup to enhance its specificity and stability. It detects CEA, neuron-specific enolase (NSE), and squamous cell carcinoma antigen (SCC) with detection limits of 0.967 ng/mL, 1.598 ng/mL, and 0.663 ng/mL, respectively. The immunogold staining method increases the sensor’s sensitivity by up to 20 times. The system, tested with samples from lung cancer patients and healthy controls, shows a strong correlation with commercial chemiluminescence assays, highlighting its potential for non-invasive, early-stage lung cancer detection. In another study [72], a miniaturized Love-SAW immunosensor is developed for the detection of CEA as part of a lung cancer screening method. The sensor is fabricated on a 36° rotated Y-cut LiTaO₃ substrate with a 2 µm SiO₂ guiding layer, operating at 165 MHz. The detection method utilizes a gold nanoparticle (AuNP)-enhanced immunoassay, where CEA antibodies are conjugated with AuNPs and undergo mass enhancement via gold staining. This approach achieves a detection limit of 1.25 ng/mL and demonstrates a strong correlation (r = 0.999) with clinical chemiluminescence assays, indicating its potential for high-accuracy, non-invasive early lung cancer detection.

#### 3.2.2. CEA Detection in Non-Exhaled Breath Condensate

A Love-SAW sensor based on an ST-cut quartz substrate operating at 120 MHz is presented in [41]. The sensor uses Au-coated IDTs and a self-assembled monolayer (SAM) of anti-CEA antibodies to detect CEA. The sensor demonstrates a sensitivity of 0.31 ng/mL and shows strong selectivity against other tumor markers like AFP and CA125. Integrated with a microfluidic chamber, the system allows real-time analysis within 30–40 min. Stability tests over 30 days revealed only an 8% decrease in performance, indicating reliable long-term functionality. In references [36,42], nanocomposite layers of polymidine doped with nanoparticles are used as sensitive layers to detect CEA by Love-SAW biosensors, and in both cases the biosensor exhibited high selectivity against other tumor markers (AFP, CA125). In [42], the biosensor is fabricated on an At-cut quartz substrate operating at 230 MHz and uses a polyimide/MXene-Au nanoparticle (PI/MXene-AuNP) nanocomposite layer, functionalized with anti-CEA antibodies through a thioglycolic acid linker. It demonstrates an impressive detection limit of 0.001 ng/mL for CEA, as well as a sensitivity of 83 Hz/ng/mL, and maintains stability for up to 75 days under periodic testing. In [36], the biosensor is fabricated on an ST-cut quartz substrate operating at 120 MHz and uses a polyimide (PI) nanocomposite layer doped with AuNP, molybdenum disulfide (MoS₂), and reduced graphene oxide (rGO). The biosensor uses the nanocomposite thin film as a bioreceptor base, which is enhanced by high-density AuNP to support anti-CEA antibody immobilization via thioglycolic acid. The experimental results reveal a low detection limit of 0.084 ng/mL for CEA and stability for up to 80 days. The study highlights the PI/AuNP–MoS₂–rGO composite’s ability to support stable acoustic wave transmission with minimal signal loss, offering a highly sensitive and durable platform for CEA detection in clinical diagnostics. In [44], a Love-SAW immunosensor is built on an ST-cut 90° X quartz substrate with a SiO_2_ wave-guiding layer of 1000 nm thickness and operates at a resonant frequency of 196 MHz. Its surface was functionalized through CEA antibody immobilization. AuNPs were bound with CEA antibodies to amplify the mass loading sensitivity. The detection range was between 0.2 and 5 ng/mL, with a limit of detection of 0.2 ng/mL. The response times ranged from approximately 10 min to 2 min. Furthermore, the sensor demonstrated low interference from the nonspecific adsorption of molecules like L-tryptophan and alpha-fetoprotein. No data about stability or reproducibility were shown.

Love-mode SAW sensors achieve enhanced detection limits and specificity for CEA through the use of nanocomposite layers, immunoassay techniques, and gold nanoparticle amplification. They demonstrate long-term stability and real-time operation, making them suitable for early cancer diagnostics across multiple sample types.

### 3.3. Alpha-Fetoprotein

Alpha-fetoprotein (AFP) is a glycoprotein primarily synthesized during fetal development by the liver and yolk sac. In adults, it is typically produced at low levels, but its expression increases in certain cancers, particularly hepatocellular carcinoma (HCC), making it a key biomarker for this type of liver cancer. AFP is often used in clinical settings to diagnose and monitor liver cancer progression. High levels of AFP in the bloodstream are indicative of malignancy in liver tissue, although some HCC patients may still have normal AFP levels, which can complicate its diagnosis [73].

In [43], the development of a novel Love-SAW aptasensor, fabricated on a ST-90° X-cut quartz with dummy fingers and based on a MoS₂/AuNPs monolayer for the detection of AFP, is presented. The sensor is capable of detecting biomarkers in serum with a dynamic range from 0.01 ng/mL to 100 ng/mL and an impressive detection limit of 4.79 pg/mL. It achieves a response time of approximately 15 min and exhibits high selectivity for AFP, a biomarker associated with liver cancer, while effectively discriminating against other biomarkers such as PSA and CEA. Additionally, the sensor retains 85% of its initial performance after 20 days and demonstrates consistent reproducibility across multiple usage cycles. In [34], a leaky SAW immunosensor for the ultrasensitive detection of AFP is developed. The device operates at a frequency of 175 MHz on a 36° YX-cut lithium tantalate (LiTaO₃) substrate, with Ti/Au-coated IDTs. The sensor uses a sandwich-type assay with MoS₂@Cu₂O-Au nanoparticles conjugated to secondary antibodies and employs gold staining to amplify the signal. The leaky SAW sensor achieves detection limits of 5.5 pg/mL with gold staining and 25 pg/mL without staining, effectively detecting AFP in human serum and saliva samples. The immunosensor shows high selectivity and a long-term stability of about five weeks when stored at 4 °C.

Using materials such as MoS₂ and AuNps, SAW sensors for AFP detection reach sensitivity levels as low as 4.79 pg/mL. These devices exhibit high selectivity against non-target biomarkers and long-term stability, enabling their application in liver cancer diagnosis and monitoring.

### 3.4. MicroRNAs

MicroRNAs (miRNAs) are small RNA molecules, typically 18–24 nucleotides long, that regulate gene expression by binding to messenger RNAs (mRNAs), leading to either degradation of the mRNA or inhibition of its translation. This process allows miRNAs to control various biological functions, including cell growth, differentiation, and apoptosis. Specific miRNAs are often upregulated or downregulated in cancer cells, and their expression profiles can serve as biomarkers for cancer detection and prognosis [74]. miR-21 is commonly overexpressed in cancers such as breast, lung, and liver cancer, where it promotes cell survival and tumor growth. miR-34, a known tumor suppressor, is frequently downregulated in many cancers, contributing to uncontrolled cell proliferation and resistance to cell death. miR-155 is elevated in cancers like lymphoma and breast cancer, and plays a role in immune response modulation and tumor progression. These miRNAs can be detected in bodily fluids such as blood, saliva, and urine, offering the potential for non-invasive cancer diagnostics.

In [27], a Love-SAW biosensor array based on a lithium tantalate (LiTaO_3_) substrate with a SiO₂ guiding layer and which operates at 200 MHz is developed. The experiments were performed with human serum spiked with three exomal miRNAs (miR-21, miR-106b, and miR-155). The sensor uses a sandwich hybridization assay with titanium dioxide (TiO₂) nanoparticles conjugated to probes, followed by photocatalytic silver staining for signal enhancement. Detection limits were as low as 0.012 pM for synthetic miRNAs and 0.21 pg/mL for exosomal miRNAs derived from MCF-7 breast cancer cells. A reference sensor was included to improve the study’s reproducibility by normalizing the sensor responses, reducing variation due to environmental factors and compensating for noise introduced by the high frequency. This configuration demonstrated high sensitivity, selectivity, and reproducibility, making it suitable for cancer biomarker detection in liquid samples.

Love-mode SAW sensors detect exomal miRNAs with limits as low as 0.012 pM. The use of titanium dioxide nanoparticles and photocatalytic silver staining enhances their sensitivity, while a reference sensor ensures reproducibility, supporting their application in non-invasive cancer diagnostics.

### 3.5. Prostate-Specific Antigen

Prostate-specific antigen (PSA) is a protein produced by the prostate gland, and is primarily used as a biomarker for prostate cancer. Elevated levels of PSA in the blood may suggest prostate cancer, although they can also indicate benign conditions like benign prostatic hyperplasia or prostatitis. PSA testing is commonly employed for screening and monitoring prostate cancer, but it has limitations, including returning false positives and negatives. Hence, it is often used in conjunction with other diagnostic tests to improve its accuracy [75].

Different studies have been carried out with SAW sensors to detect this biomarker. Thus, a Love wave sensor utilizing hydrophilic MIP layers to detect prostate-specific membrane antigen (PSMA) was fabricated. The MIP is synthesized via reversible addition-fragmentation chain transfer (RAFT) polymerization, enhancing its specificity and minimizing non-specific protein interactions. A 160 MHz Love wave is generated on a piezoelectric quartz substrate with a SiO₂ guiding layer to concentrate acoustic energy and to improve the sensor’s sensitivity. The sensor detects PSMA with a limit of 0.013 ng/mL and shows high stability and reversibility that are comparable to ELISA assays. This approach enables detection in complex media like mouse serum, making it suitable for early prostate cancer diagnostics [37]. In [46], another cost-effective SAW biosensor is designed for early prostate cancer detection by quantifying PSA levels in biological samples. The device utilizes SH-SAWs on an ST-cut quartz substrate to detect mass loading changes caused by PSA binding, with phase detection as the primary measurement method. A simplified driver circuit generates a 16.9 MHz square wave, while phase shifts are extracted using a low-frequency down-sampling technique (100 kHz), reducing the hardware complexity and costs. Despite using a 1-bit analog-to-digital converter (ADC), this down-sampling method ensures phase accuracy with errors as low as 1% and a minimum phase delay of 0.3 ns. This streamlined design demonstrates that accurate PSA quantification is achievable with sufficient samples, making it suitable for single-chip integration and potential point-of-care applications. In [48], a device is fabricated which operates on a ZnO-coated Si substrate and utilizes both Rayleigh and Sezawa wave modes. When the ZnO film is thinner than 2.8 µm, the Rayleigh wave dominates, while thicker films (>2.8 µm) facilitate the Sezawa wave mode. The biosensor, operating around 130 MHz in the Sezawa mode, uses a cystamine SAM and anti-PSA antibody on gold electrodes to achieve linear frequency shifts for PSA concentrations ranging from 2 to 20,000 ng/mL. By combining SAW streaming effects with the advantages of Sezawa waves, the system enables efficient, label-free PSA detection with a minimal sample volume, offering a real-time, portable tool for prostate cancer diagnostics.

Advanced designs incorporating molecularly imprinted polymers (MIPs) and self-assembled monolayers (SAMs) enable the detection of PSA and PSMA with limits as low as 0.013 ng/mL. Innovations such as simplified circuits and Sezawa wave modes support real-time, label-free detection, making these sensors suitable for compact, point-of-care applications.

### 3.6. Other Protein Biomarkers

Numerous proteins that serve as cancer biomarkers have been successfully detected using SAW sensors, including:

#### 3.6.1. Epidermal Growth Factor

Epidermal growth factor (EGF) is a key protein involved in the regulation of cell growth, proliferation, and survival. It binds to the epidermal growth factor receptor (EGFR), which is a receptor tyrosine kinase. EGFR is commonly overexpressed or mutated in many cancers, including non-small-cell lung cancer, colorectal cancer, glioblastoma, and breast cancer, where it promotes oncogenic signaling pathways that facilitate cancer cell proliferation. As biomarkers of cancer, EGF and EGFR have significant roles. The overexpression or mutations of EGFR are commonly associated with several types of cancer, including non-small cell lung cancer, colorectal cancer, and glioblastomas. These mutations often lead to the continuous activation of signaling pathways that promote cancer cell proliferation and resistance to apoptosis (programmed cell death) [76].

In [31], a SH-SAW biosensor was developed for the detection of epidermal growth factor (EFG) using 3-aminopropyltriethoxysilane (APTES) and glutaraldehyde films as functional layers. The sensor operates at a frequency of 121.6 MHz on a 36° Y-X LiTaO_3_ substrate with a SiO_2_ guiding layer. EGF is captured through surface functionalization with either APTES or glutaraldehyde. Experimental findings reveal frequency shifts proportional to EGF concentrations ranging from 0.2 to 5 ng/mL, with the glutaraldehyde film demonstrating superior sensitivity at 1.709 kHz/(ng/mL), compared to 0.641 kHz/(ng/mL) for the APTES film [31]. A semi-empirical model, modified from the Sauerbrey equation, enables the direct conversion of the frequency shift to the analyte concentration. The biosensor shows promise for real-time, label-free cancer biomarker detection in biofluids.

#### 3.6.2. C-Reactive Protein, Lipoprotein (a), and Apolipoprotein B

In [33], a SH-SAW sensor system operating at 250 MHz was constructed using a dual-channel delay line configuration on a 36° Y-cut, 90° X-propagated quartz substrate with a thickness of 0.5 mm. This SH-SAW sensor demonstrated the capability to detect biological markers, including C-reactive protein (CRP), lipoprotein (a) (Lp(a)), and apolipoprotein B (ApoB), which are associated with inflammatory processes linked to cancer. The sensor achieved high sensitivity, with detection limits for CRP spanning from 1.9 to 118 µg/mL and a limit of detection (LoD) of 390 ng/mL. For Lp(a), it showed a detection range from 83 to 1402 µg/mL. The system also provided rapid responses, with detection times as short as 3 min.

#### 3.6.3. Human Mammaglobin

Human Mammaglobin (hMAM) is a protein primarily found in human breast tissue. It is known as a molecular marker and is widely used in oncology, particularly for the detection and monitoring of breast cancer.

In [39], a CMOS-integrated SAW (147–150 MHz Rayleigh waves) biosensor is fabricated for the detection of hMAM, a breast cancer biomarker, using a streptavidin/biotin-based immunoassay. The SAW device operates on a 0.5 μm CMOS platform with ZnO as the piezoelectric layer and a gold coating for functionalization, enabling the binding of hMAM-specific antibodies. Testing demonstrated a frequency sensitivity of 8.704 pg/Hz and a mass sensitivity of 2810.25 m²/kg. Its selectivity was validated against bovine serum albumin, confirming specific hMAM detection. The device shows potential for low-cost, scalable cancer biomarker detection with high sensitivity in a miniaturized format.

#### 3.6.4. Cancer Antigen 125

Cancer antigen 125 (CA125) is a glycoprotein found on the surface of many cells, and its levels are often elevated in the blood of patients with ovarian cancer. However, it is important to note that elevated CA125 levels are not exclusive to ovarian cancer and can be found in conditions such as endometriosis, pelvic inflammatory disease (PID), menstruation, liver disease, and other cancers like lung, breast, and pancreatic cancer. It is most commonly used to track the effectiveness of treatment in patients with known ovarian cancer, particularly after surgery or chemotherapy and in conjunction with imaging and other tests to assess the possibility of ovarian cancer.

Reference [30] presents a Rayleigh-mode SAW (SAW fabricated with ST-cut quartz) immunosensor for detecting carcinoma antigen 125 (CA125), an ovarian cancer biomarker. It operates at 203.5 MHz in a delay-line configuration. The sensor employs a chemically vapor-deposited graphite tube decorated with gold nanoparticles on the inside as both the fluidic channel and sensing element. The graphene–AuNP structure enhances the sensor’s sensitivity by trapping CA125 antibodies within the fluidic channel. It demonstrated high sensitivity with a wide linear response from 0.01 to 300 mU/mL and a detection limit of 0.00371 mU/mL. The device showed excellent specificity against other biomarkers and retained stability over extended use, supporting its potential for use in biomedical diagnostics.

#### 3.6.5. B-Cell Lymphoma 2

B-cell lymphoma 2 (Bcl-2) is a key regulator of apoptosis (programmed cell death). It belongs to a family of proteins that control the balance between cell survival and death, playing a crucial role in maintaining cellular homeostasis by deciding whether a cell should live or die.

In [32], a SH-SAW biosensor was developed on an ST-cut quartz substrate for the detection of Bcl-2 protein, a urinary biomarker associated with early ovarian cancer. Operating at a frequency of 16.8 MHz, the sensor employs a delay line structure with microfabricated IDTs and a functionalized delay path. The detection mechanism relies on an anti-Bcl-2 monoclonal antibody layer to capture Bcl-2. The sensor achieves a sensitivity of 0.5 ng/mL, exhibiting a linear frequency response to increasing Bcl-2 concentrations. To minimize non-specific adsorption, the surface is functionalized with SAMs protein A/G, and Pluronic F127. The sensor demonstrates selective and repeatable detection over up to 10 cycles, highlighting its potential as a non-invasive tool for ovarian cancer screening.

#### 3.6.6. Streptavidin

Streptavidin is a protein sourced from the bacterium *Streptomyces avidinii*, known for its exceptionally high and specific affinity for biotin (vitamin B7). This interaction represents one of the strongest non-covalent bonds found in nature. While it is not a cancer biomarker itself, it plays a crucial role in cancer biomarker detection when used in conjunction with biotin-labeled probes.

In [40], A Rayleigh SAW microfluidics-based lab-on-a-chip (LoC) device was developed for biosensing applications, and was specifically tailored for the detection of biomolecules in complex media. The device incorporates four nanoscale SAW resonators operating at 1.2 GHz, along with a microfluidic system that facilitates multiplexed measurements and fluid mixing. It is constructed on 128° X-rotated Y-cut lithium niobate (LN) wafers. A biotin-polyethylene glycol (bPEG) coating is applied to the surface to prevent fouling and improve the sensor’s selectivity by inhibiting non-specific binding. While streptavidin (SA) is not directly related to cancer, its stable, specific binding to biotin makes it a valuable component in biosensing. When combined with biotin-labeled probes, SA can effectively assist in detecting cancer biomarkers. The device achieved a sensitivity threshold of 290 pM for SA, even in the presence of high concentrations of bovine serum albumin (BSA), a crucial compound for sensing applications in biological fluids where interfering proteins are prevalent. In [50], a Rayleigh SAW resonator biosensor was developed utilizing both positive and negative reflectors to improve the sensor’s biomolecule detection in liquid samples after drying. Operating at a high frequency of 1.285 GHz on a lithium niobate (LiNbO₃) substrate, the device incorporates IDTs without a guiding layer, enabling the direct binding of molecules to the resonant surface to optimize its sensitivity. The sensor demonstrates a limit of detection (LoD) of 104 pM for biotin-streptavidin and a normalized mass sensitivity of −296 m²/kg. The inclusion of positive and negative reflectors helps to concentrate wave energy within the sensitive area, enhancing both the detection stability and the dynamic range of the sensor.

#### 3.6.7. Alfa-Glycosidase

Alpha-glycosidase is an enzyme that plays a key role in the digestion of carbohydrates. It is involved in the breakdown of complex carbohydrates (such as starch and disaccharides) into simpler sugars like glucose. This enzyme is found primarily in the small intestine and is essential for the absorption of sugars from the digestive tract. Such enzymes could participate in processes related to carbohydrate metabolism, oxidative stress, or glycation product breakdown. α-Glycosidase is an enzyme involved in carbohydrate metabolism, and while not a primary cancer biomarker, its altered activity may reflect metabolic changes associated with certain cancers.

In [26], a high-frequency Rayleigh surface acoustic wave lab-on-a-chip biosensor operating at 740 MHz for the detection of active α-glycosidase is developed. The device, fabricated on a LiNbO_3_ substrate, is functionalized with a specially designed probe molecule, 7-mercapto-1-eptyl-D-maltoside, which mimics a substrate for α-glycosidase. The sensor was tested in aqueous samples with α-glycosidase concentrations from 0.15 to 150 U/mL, achieving a detectable signal within this range and showing strong sensitivity to active enzyme concentrations up to 15 U/mL. To ensure specificity, the sensor was also tested with acarbose, an enzyme inhibitor, resulting in an 80% signal decrease in its presence.

SAW sensors reliably detect EGF, CRP, Lp(a), ApoB, hMAM, CA125, Bcl-2, and alpha-glycosidase, each having distinct properties. For example, CMOS-integrated sensors for hMAM provide high sensitivity in miniaturized formats, while Rayleigh-mode sensors for CA125 achieve a wide detection range. These systems address various diagnostic needs in oncology.

### 3.7. Cancerous Cells

Cancer cells are cells that grow and divide uncontrollably, ignoring the normal regulatory mechanisms of the body. Unlike healthy cells, they can invade surrounding tissues and spread to other parts of the body (a process known as metastasis). These cells often result from genetic mutations that disrupt normal cell functions, such as growth, repair, and programmed cell death (apoptosis).

Currently, methods such as MTT assay, flow cytometry, and Ki67 staining are used to assess cell growth or proliferation in 2D cultures. In 3D cultures, measurements typically involve endpoint procedures like trypsinization, trypan blue staining, and quantification. However, there is a significant need for non-invasive, contact-free techniques to monitor the growth or proliferation of cells over time, particularly for 3D tumor spheroid models and stem cell regeneration studies. Using SAW sensors could provide an innovative alternative to these existing approaches [47]. In [47], a SH-SAW biosensor was developed for quantifying cell growth in both 2D and 3D cultures. The SH-SAW device, built on a 36° Y-cut lithium tantalate (LiTaO₃) substrate with a 200 nm ZnO coating, operates at 14.05 MHz and incorporates IDTs and a polydimethylsiloxane (PDMS) well to support cell cultures. The ZnO layer enhances the sensor’s sensitivity by amplifying frequency shifts caused by cell mass loading, providing up to four times greater sensitivity compared to an uncoated sensor. The device successfully detected cell concentrations ranging from 6250 to 50,000 cells per 100 µL for both cancerous (A549) and non-cancerous (RAW264.7) cells. Additionally, the SH-SAW biosensor monitored the cell growth over eight days of A549 spheroids cultured on a 3D nanofiber scaffold, showing a linear frequency shift that correlated with the tumor spheroid expansion over time. This SH-SAW biosensor provides a real-time, non-invasive tool for studying cell proliferation in 3D models, making it a valuable asset for cancer research and regenerative medicine applications. In [77], a SAW biosensor configured as a delay line was developed on an X-cut lithium niobate (LiNbO₃) substrate, operating at a center frequency of 10.3 MHz, for the detection and differentiation of primary (HT-29) and metastatic (SW-48) colon cancer cells. Significant resonance peak shifts were observed, especially with SW-48 cells, which exhibited higher negative surface charges compared to HT-29 cells. This variation primarily influenced the wave through electroacoustic interactions. These findings highlight the potential for distinguishing cell types based on their electroacoustic profiles, offering a promising approach for diagnosing cancer progression. In [53], a SH-SAW biosensor was developed on a Y36°-X lithium tantalate (LiTaO₃) substrate to detect antigen-specific cells, such as T cells, in liquid environments. Operating at 122.5 MHz with a SiO₂ waveguiding layer, the sensor was functionalized with CD3 antibodies to selectively capture Jurkat cells from a mixed-cell suspension. A liquid microchamber integrated with a peristaltic pump facilitated continuous flow, enabling rapid, label-free cell detection with a sensitivity of 1000 cells/mL and a frequency shift of 280 Hz. This SH-SAW platform offers a rapid and efficient method for cell detection, making it highly relevant for monitoring immune and cancer cells. In [78], an SH-SAW sensor was developed for the rapid detection and purification of tumor cells (TCs), specifically colorectal adenocarcinoma HCT-8 cells, from biofluids. The sensor operates at 122.5 MHz on a 36° Y-X LiTaO₃ substrate, incorporating a 4 µm SiO₂ guiding layer to facilitate wave propagation in liquid environments. The device is equipped with Au/Cr IDTs, which were fabricated using photolithography with 50 finger pairs and a 34 µm periodicity. It demonstrates a mass sensitivity of 4 Hz/ng, enabling the selective detection of TCs through a microfluidic system. Stability testing revealed a minimal frequency drift under varying humidity and temperature conditions, while a dual-delay line configuration enhanced the sensor’s selectivity by reducing acoustoelectric interference. This SH-SAW biosensor, combined with a micro-incubation feature, supports the non-invasive, label-free detection of cancer cells in clinical applications. Additionally, in [24], a hybrid sensor integrating SAWs and dielectrophoresis (DEP) was developed for trapping and detecting cells, including white blood cells (WBCs), RPMI, and U87 cells. The device utilizes a Love wave-based SAW mechanism on a lithium niobate (LiNbO₃) substrate with a zinc oxide (ZnO) waveguide layer, an operates at approximately 142 MHz. The DEP force, used in place of a sacrificial layer, traps cells, making the sensor reusable and versatile for cell detection based on their dielectric properties. This configuration enhances the sensor’s sensitivity to 5.6 × 10^−5^ dB/cell/mL (or 2.13 Hz/cell/mL), providing stable and selective detection. Changes in the resonance frequency and amplitude allow differentiation between natural and cancerous cells from the brain, intestine, and breast, offering a reusable platform for detecting and releasing various cell types.

SH-SAW sensors offer the real-time, non-invasive monitoring of cell growth in 2D and 3D cultures, distinguishing between primary and metastatic colon cancer cells based on electroacoustic interactions. Hybrid SAW–DEP sensors further enable the reusable detection of various cell types by leveraging their dielectric properties, highlighting their adaptability for cancer research.

### 3.8. Nucleosides

Nucleosides are organic molecules that consist of two components: a nitrogenous base (which can be a purine or pyrimidine) and a sugar molecule (either ribose or deoxyribose). They are the building blocks of nucleotides, which are essential for the formation of DNA and RNA. Unlike nucleotides, nucleosides do not have phosphate groups attached to them.

Nucleosides can serve as cancer biomarkers due to their altered levels in biological fluids (e.g., blood or urine) under pathological conditions, including cancer. Changes in nucleoside metabolism often occur in cancer cells due to increased proliferation, DNA synthesis, and turnover, making them potential indicators of cancer activity [79].

In [55], a Love-SAW sensor is described that utilizes MIPs for the detection of nucleosides, such as adenosine-5′-monophosphate (5′-AMP) and pseudouridine (Pseu), which serve as cancer biomarkers. The sensor, developed on an AT-cut quartz substrate with a 4 µm SiO₂ guiding layer, operates at 118 MHz. The MIP layer is specifically imprinted to bind 5′-AMP and Pseu, enabling selective interaction with these nucleosides. The device is integrated with a microfluidic system for real-time monitoring. The sensor achieves a detection limit of 5 ppm for 5′-AMP, with frequency shifts of about 150 Hz per injection, highlighting its potential for non-invasive cancer biomarker analysis in liquid samples for monitoring treatment efficacy. Similarly, in [56], another Love-wave sensor coated with a MIP is developed to detect AMP as a representative nucleoside linked to cancer biomarkers. This sensor operates on an AT-cut quartz substrate at 117 MHz with a 4 µm SiO₂ guiding layer and uses a porous AMP-imprinted polymer coating for selective AMP binding. In aqueous media, the sensor demonstrates a frequency shift of approximately 137 Hz per ppm for AMP concentrations as low as 6 ppm, proving its high sensitivity. The sensor’s reproducibility across measurements has been confirmed, with there being plans to extend its application to detect specific cancer-related nucleosides. In [57], a love-wave surface acoustic wave sensor is presented, coated with a molecularly imprinted polymer (MIP) layer, that is designed for detecting AMP as a model for colorectal cancer biomarkers. The sensor, built on an AT-cut quartz substrate with a 4.5 µm SiO₂ guiding layer, operates at 117 MHz. The MIP layer, specifically designed to recognize AMP, demonstrated selectivity and sensitivity, with frequency shifts of up to −2.5 kHz in response to 2126 ppm of ethanol vapor, which were observed when comparing pre- and post-template extraction stages. The response was found to increase with thicker MIP films (200 nm vs. 600 nm), indicating the sensor’s potential for sensitive and selective biomarker detection in gas-phase sensing applications relevant to cancer monitoring.

This body of literature demonstrates the development of similar devices for detecting AMP, underscoring their potential in cancer diagnostics.

Love-mode SAW sensors detect nucleosides like AMP and pseudouridine with limits as low as 5 ppm, using molecularly imprinted polymers to obtain high selectivity. These sensors excel in liquid- and gas-phase applications, with microfluidic integration enabling real-time biomarker analysis for cancer monitoring.

### 3.9. Circulating Tumor Cells

Circulating tumor cells (CTCs) are cancer cells that break away from the primary tumor and travel through the bloodstream. These cells play an essential role in metastasis, which is the process by which cancer spreads to other organs. While most CTCs are unable to survive in the blood due to the hostile environment, a small fraction can evade immune defenses and form secondary tumors in distant sites. Detecting and analyzing these cells provides crucial information about cancer’s progression, its metastatic capabilities, and its response to treatments [80].

In [25], a leaky SAW aptasensor array is presented which is designed for the label-free, high-sensitivity detection of CTCs, specifically MCF-7 breast cancer cells. The sensor operates on a 36° YX-cut lithium tantalate (LiTaO₃) substrate at 100 MHz, with aptamers targeting MUC1 proteins on the cell surface for the capture of MCF-7 cells. The array consists of five detection sensors and one reference sensor, each with independent oscillation circuits to ensure stability. This setup offers a broad linear detection range from 100 to 10⁷ cells/mL, with a detection limit as low as 32 cells/mL. The sensor also demonstrates excellent specificity, distinguishing MCF-7 cells from non-target cells, and retains 90% of its performance after 10 regeneration cycles, highlighting its potential for clinical cancer diagnostics.

Leaky SAW aptasensors detect MCF-7 breast cancer cells with a detection limit of 32 cells/mL, using MUC1-targeting aptamers for high specificity. Independent oscillation circuits ensure stability, and their regeneration capabilities support reliable clinical diagnostics.

### 3.10. Genes

Human epidermal growth factor receptor 2 (HER2/neu) is a gene that plays a key role in the development of certain types of cancer, especially breast cancer. It encodes a protein that is involved in cell growth and division. When the HER2 gene is amplified (i.e., produces too many copies) or the HER2 protein is overexpressed on the surface of cells, it can promote cancerous growth. This is commonly seen in some breast cancers, referred to as HER2-positive breast cancer.

In [81], a label-free SH-SAW biosensor for detecting HER-2/neu, a breast cancer biomarker, is presented. The sensor is based on a 36° YX-LiTaO₃ substrate operating at 428.5 MHz. It employs neutravidin and biotinylated protein A linkers for site-directed immobilization of anti-HER-2 antibodies, enhancing the sensor’s signal response and achieving a detection limit of 10 ng/mL. The sensor is fabricated with a parylene C layer to ensure chemical homogeneity and is integrated with a microfluidic flow cell, enabling real-time detection under low-flow conditions. Stability tests using PBS and selectivity evaluations against bovine serum albumin demonstrated its strong potential for non-invasive clinical breast cancer diagnostics [81]. BRCA1, PTCH, and p53 are all crucial genes that play significant roles in cell regulation, DNA repair, and tumor suppression, and mutations in these genes are associated with various types of cancer. Mutations in BRCA1 are most famously linked to hereditary breast and ovarian cancers, significantly increasing the risk of developing these cancers. Mutations in PTCH are associated with Basal Cell Nevus Syndrome (Gorlin syndrome), which increases the risk of developing basal cell carcinoma and other tumors like medulloblastoma. Mutations in p53 are among the most common genetic alterations found in human cancers, including lung, colon, and breast cancers. In [28], a Love-SAW sensor is presented for the detection of single-nucleotide mutations in cancer-associated genes, including BRCA1, PTCH, and p53. Operating at 148 MHz on an ST-cut quartz substrate with a carboxymethylated dextran (CMD) layer, the sensor utilizes a streptavidin–biotin system for the immobilization of DNA probes, enabling the use of hybridization assays. The sensor differentiates between mutant and wild-type sequences by analyzing mass and viscosity changes utilizing association/dissociation kinetics, with a detection sensitivity of 10 nM DNA concentrations. The system allows for multiple binding cycles with minimal signal degradation, providing a rapid, label-free method for mutation detection which holds potential for clinical genetic cancer screening applications.

SH-SAW sensors for HER2/neu provide real-time detection with a 10 ng/mL limit, while Love-wave sensors detect mutations in BRCA1, PTCH, and p53 with a 10 nM sensitivity. These sensors differentiate mutant from wild-type DNA, offering valuable tools for genetic cancer screening.

Table 3 shows a summary of the biomarkers that have been detected by SAW biosensors in studies this review. For each biomarker, the concentration range detected and the type of cancer it is associated with are given.

## 4. Future Trends

From the perspective of traditional biomarker detection methods, techniques such as enzyme-linked immunosorbent assays (ELISAs) and polymerase chain reaction (PCR) are constrained by significant technological limitations, including slow detection times and the high cost of reagents for each assay. These methods are also labor-intensive and not conducive to continuous patient monitoring during treatment. Additionally, given that cancer is a multifactorial disease involving complex cellular events and multiple molecular interactions, the simultaneous detection of various biomarkers is essential for accurate diagnosis and prognosis. Moreover, the detection of biomarkers such as VOCs by traditional techniques, such as gas chromatography-mass spectrometry (GC-MS), is also subject to similar limitations as those observed with ELISAs and PCR, including long analysis times and high operational costs.

The same happens for cancerous cell detection by imaging techniques (CT, SPECT, MRI, and PET) and biopsies, which are also very expensive in terms of their operational costs and acquisition costs.

Due to the limitations of the aforementioned techniques, the development of advanced biosensing devices for clinical applications such as detecting cancer cells and their biomarkers has become a growing area of research. This has served as the driving motivation behind this review on acoustic biosensors for cancer detection, with the objective of assessing the current advancements in the field and exploring potential avenues for enhancement in future research. The selection of SAW biosensors is based on the advantages they offer for potential clinical applications, such as label-free detection, real-time operation, and compatibility with miniaturized and portable systems, making them promising tools for non-invasive cancer diagnosis.

However, the main challenges in the use of chemical sensors and biosensors are their stability, repeatability, and reproducibility. These practical issues hinder the application of these devices outside laboratory settings. Advances in sensing layer immobilization should address these challenges, and future research should focus on reporting sensor characteristics in this area. As emphasized in this review, despite the significant amount of research on SAW biosensors, much of it has been directed towards environmental monitoring and food safety, rather than cancer diagnostics. Translating advancements in SAW biosensor technology and in nanomaterial technology into the realm of cancer diagnostics could unlock significant innovations and breakthroughs. Thus, in addition to their established role in detecting complex biomolecules, SAW sensors are also capable of detecting low-molecular-weight VOCs in breath, which are increasingly recognized as potential biomarkers for a variety of diseases, including cancer in its early stages. However, as pointed out in this paper, this area remains underexplored in the context of cancer detection. The limited literature available on the detection of VOCs as biomarkers using SAW sensors is primarily focused on lung cancer diagnostics. For detecting VOCs, SAW sensors can incorporate diverse sensing materials such as metal oxides [82], stationary phases [83], room temperature ionic liquids (RTILs) [84], and nanostructured materials [85]. Stationary phases, commonly used in gas chromatography, can be integrated into SAW devices as sensitive layers, improving their specificity for particular VOCs. Polymers like polydimethylsiloxane (PDMS), polyethylene glycol (PEG), and other materials can be coated onto the SAW sensor surface, where they interact with VOC molecules through absorption or adsorption processes. By selecting or engineering polymers with specific chemical affinities, the sensitivity and selectivity of SAW sensors can be significantly enhanced for targeted VOCs. Additionally, the incorporation of lipid bilayers, which replicate the natural environment of cell membranes, can facilitate the integration of membrane proteins or receptors into the sensor surface. While this strategy has been successfully applied in QCM sensors, it remains relatively unexplored in SAW devices.

Another line of innovation arises from the advancement of more sophisticated systems, including flexible SAW sensors [86,87], which can be incorporated into wearable devices for continuous, real-time health monitoring. These sensors offer the potential for non-invasive detection and analysis, enabling applications such as early disease diagnosis, personalized health tracking, and the monitoring of environmental exposure to harmful substances.

Finally, an additional cutting-edge direction for future research is the integration of SAW sensors with microfluidic technology. These devices are particularly well-suited for integration with microfluidic systems, creating powerful platforms for cancer diagnostics. Their ability to manipulate fluids and particles at the micro scale enables precise control over sample handling, which is essential for lab-on-a-chip technologies. While some studies have begun exploring this integration [88,89], there is significant potential for further development. Advancing this field could lead to highly sensitive, rapid, and portable diagnostic tools, enhancing early cancer detection and personalized medicine. If, in addition, biomarker data are integrated with artificial intelligence techniques, this is expected to enhance diagnostics and therapies, leading to more efficient and effective healthcare solutions.

## 5. Conclusions

There is a dire need for biosensors that can rapidly analyze cellular modifications to identify biomarkers associated with cancer in order to enhance the prognosis and therapy options regarding cancer, especially at the early stage of disease.

SAW sensors have evolved into a promising tool for cancer biomarker detection, combining high sensitivity, real-time operation, label-free detection, and versatility in detecting a wide range of analytes. This review has outlined how specific configurations, such as Love-wave and SH-SAW devices, are particularly suited to the challenges of detecting biomarkers in both gaseous and liquid environments, including VOCs in exhaled breath and biomolecules in bodily fluids. Love-SAW and SH-SAW sensors are overrepresented in the research, particularly Love-SAW sensors, as they exhibit higher sensitivities due to the wave mode used. Table 3 shows a summary of the biomarkers that have been studied in the field of SAW biosensors. While the detected biomarkers do not provide a comprehensive review of all cancer biomarkers, they encompass all the research conducted on SAW biosensors to the best of our knowledge.

Nevertheless, significant challenges must be addressed before SAW sensors can become a standard tool in cancer diagnostics. Current research directions include optimizing the sensitivity of sensors for specific biomarkers, mitigating the effects of environmental variables like humidity, and validating their clinical performance across diverse patient populations, primarily for VOC biomarkers. Furthermore, while recent advancements in piezoelectric materials, guiding layers, and functionalization techniques have improved sensor performance, these innovations often increase the manufacturing complexity and cost.

Future research should prioritize the integration of SAW sensors into hybrid diagnostic systems that combine complementary technologies, such as gas chromatography or MOS sensor technology for VOC sensing, to enhance their detection specificity, sensitivity for low concentrations, stability, reproducibility, and robustness. Additionally, efforts to miniaturize and standardize SAW devices for widespread clinical use will be essential to bridging the gap between laboratory research and real-world applications.

In conclusion, while SAW sensors still face technical and practical hurdles, their potential to revolutionize cancer diagnostics through non-invasive, scalable, and real-time detection methods is undeniable. Further innovation in sensor design and validation in clinical settings will determine their ultimate impact in addressing the global burden of cancer. This review envisions a promising future for biomarkers detected by SAW sensors, driven by technological innovations that will enhance early cancer detection and treatment personalization, significantly contributing to the advancement of modern medicine.

## Figures and Tables

**Table 1:** Comparison of SAW biosensors with traditional analytical methods to detect cancer. In bold the data related to SAW biosensors, Green, yellow, and orange denoting scale in each category (column) from better to worse.

Analyte Type	Technique	Sensitivity	Specificity	Complexity	Speed	Cost	Early Detection Capability	Analyte Range
VOCs	GC-MS	High	High	High	Slow	High	Moderate	Broad
	**SAW**	**High**	**Moderate**	**Low**	**Fast**	**Low**	**High**	**Design Limited**
Proteins	ELISA	High	High	Moderate	Moderate	Moderate	High	Design Limited
	**SAW**	**High**	**High**	**Low**	**Fast**	**Low**	**High**	**Design Limited**
Genetic Molecules	PCR	High	High	High	Moderate	High	High	Design Limited
	**SAW**	**High**	**High**	**Low**	**Fast**	**Low**	**High**	**Design Limited**
Cells	Cytology	Moderate	Moderate	High	Slow	Moderate	Low	Limited
	Imaging	Moderate	Moderate	High	Moderate	High	Low	Limited
	Endoscopy	Moderate	Moderate	High	Moderate	High	Low	Limited
	**SAW**	**High**	**High**	**Low**	**Fast**	**Low**	**High**	**Design Limited**

**Table 2:** Types of SAW devices used in cancer biomarker detection.

Type	Wave Propagation	Sensitivity	Energy Loss	Operating Frequency	Piezoelectric Material	Guiding Layer	Special Requirements	Reference
Rayleigh	Surface, elliptical particle motion	High sensitivity to surface changes	Significant in liquids	150 MHz–1.2 GHz MHz	Quartz, LiTaO_3_, LiNbO_3_, ZnO		Not suitable for liquids	[26,30,39,40]
Love	Horizontally polarized, surface guided	High sensitivity in liquids, surface-sensitive	Minimal in liquids	5 MHz–230 MHz	Quartz, ZnO, LiTaO₃, LiNbO₃	Polyimide, PMMA, Polypyrrole, SiO_2_, MoS_2_, ZnO, CMD	Requires waveguide layer	[22,24,27,36,37,41,42,43,44,45]
SH-SAW	Shear horizontal, surface	Sensitive to liquid samples, surface perturbations	Minimal in liquids	17 MHz–250 MHz	LiTaO_3_, LiNbO_3_, Quartz	SiO_2_, ZnO	Suitable for liquids due to horizontal displacement	[31,33,46,47]
Leaky-SAW	Surface, with energy leakage into bulk	Sensitive to liquid samples, moderate sensitivity	Leakage into bulk	175 MHz–240 MHz	LiTaO_3_, LiNbO_3_	SiO_2_	Requires metal film to mitigate leakage	[25,34]
Sezawa	High order Rayleigh guided wave	High sensitivity to surface changes	Significant in liquids	130 HMz	ZnO	Si	Guide layer with wave speed lower than the substrate	[48]

**Table 3:** Cancer biomarkers detected by different SAW biosensor types.

Type of Biomarker	Cancer Associated	Concentration Ranges	Type of Sensors
Volatile Organic Compounds (VOCs)	Lung, Gastrointestinal	3 ppb to 0.2 ppm	Love-SAW, Rayleigh SAW
Carcinoembryonic Antigen (CEA)	Colorectal	0.1 ng/mL to 16 ng/mL	Love-SAW
Alpha-fetoprotein (AFP)	Liver	0.0055 ng/mL to 100 ng/mL	Love-SAW, Leaky-SAW
MicroRNA (miRNA)	Breast, Lymphoma, Liver, Lung	0.0021 ng/mL to 0.00012 ng/mL	Love-SAW
Prostate-Specific Antigen (PSA)	Prostate	0.013 ng/mL to 20,000 ng/mL	Love-SAW, SH-SAW, Sezawa-SAW
Other Proteins	Epidermal, Breast, Ovarian, Colorectal, Pancreatic	0.2 ng/mL to 1.402 ± 10^6^ ng/mL	SH-SAW, Rayleigh-SAW
Cancer Cells	Lung, Colorectal, Brain, Breast, Immune system	1000 cells/mL to 5 × 10^7^ cells/mL	SH-SAW, Rayleigh-SAW
Nucleosides	Colorectal	5 ppm to 200 ppm	Love-SAW
Circulating Tumor Cells (CTCs)	Breast	100 to 10^7^ cells/mL	Leaky-SAW
Genes	Breast	10 nM DNA	Love-SAW, SH-Love

## Data Availability

Data sharing is not applicable.

## References

[1] World Health Organiation Cancer Key Facts. https://www.who.int/news-room/fact-sheets/detail/cancer.

[2] Manhas N., Kumar L.S., Adimule V., Kendrekar P., Adimule V., Hurst T. (2023). Early-Stage Diagnosis of Breast Cancer: Amelioration in Approaches. Drug and Therapy Development for Triple Negative Breast Cancer.

[3] Kokabi M., Tahir M.N., Singh D., Javanmard M. (2023). Advancing Healthcare: Synergizing Biosensors and Machine Learning for Early Cancer Diagnosis. Biosensors.

[4] Azab M.Y., Hameed M.F.O., Obayya S.S.A. (2023). Overview of Optical Biosensors for Early Cancer Detection: Fundamentals, Applications and Future Perspectives. Biology.

[5] Sarhadi V.K., Armengol G. (2022). Molecular Biomarkers in Cancer. Biomolecules.

[6] Khan H., Shah M.R., Barek J., Malik M.I. (2023). Cancer Biomarkers and Their Biosensors: A Comprehensive Review. TrAC Trends Anal. Chem..

[7] Sarkar S., Hazra S., Patra S., Gogoi M. (2024). Biosensors for Cancer Detection: A Review. TrAC Trends Anal. Chem..

[8] Das S., Dey M.K., Devireddy R., Gartia M.R. (2024). Biomarkers in Cancer Detection, Diagnosis, and Prognosis. Sensors.

[9] Abdul Wahab M.R., Palaniyandi T., Viswanathan S., Baskar G., Surendran H., Gangadharan S.G.D., Sugumaran A., Sivaji A., Kaliamoorthy S., Kumarasamy S. (2024). Biomarker-Specific Biosensors Revolutionise Breast Cancer Diagnosis. Clin. Chim. Acta.

[10] Rayleigh L. (1885). On Waves Propagated along the Plane Surface of an Elastic Solid. Proc. Lond. Math. Soc..

[11] Tang Z., Wu W., Yang P., Luo J., Fu C., Han J.-C., Zhou Y., Wang L., Wu Y., Huang Y. (2024). A Review of Surface Acoustic Wave Sensors: Mechanisms, Stability and Future Prospects. Sens. Rev..

[12] Zhang J., Zhang X., Wei X., Xue Y., Wan H., Wang P. (2021). Recent Advances in Acoustic Wave Biosensors for the Detection of Disease-Related Biomarkers: A Review. Anal. Chim. Acta.

[13] Mandal D., Banerjee S. (2022). Surface Acoustic Wave (SAW) Sensors: Physics, Materials, and Applications. Sensors.

[14] Zida S.I., Lin Y., Khung Y.L. (2021). Current Trends on Surface Acoustic Wave Biosensors. Adv. Mater. Technol..

[15] Li W., Liu H.-Y., Jia Z.-R., Qiao P.-P., Pi X.-T., Chen J., Deng L.-H. (2014). Advances in the Early Detection of Lung Cancer Using Analysis of Volatile Organic Compounds: From Imaging to Sensors. Asian Pac. J. Cancer Prev..

[16] Oyerinde A.S., Selvaraju V., Babu J.R., Geetha T. (2023). Potential Role of Oxidative Stress in the Production of Volatile Organic Compounds in Obesity. Antioxidants.

[17] Fourati N., Attia G., Khaoulani S., Zerrouki C., Lieberzeit P. (2024). Applications and Recent Trends in Surface Acoustic Wave Biosensors. Piezoelectric Sensors.

[18] Gouda M., Ghazzawy H.S., Alqahtani N., Li X. (2023). The Recent Development of Acoustic Sensors as Effective Chemical Detecting Tools for Biological Cells and Their Bioactivities. Molecules.

[19] White R.M., Voltmer F.W. (1965). Direct Piezoelectric Coupling to Surface Elastic Waves. Appl. Phys. Lett..

[20] Yang Y., Dejous C., Hallil H. (2022). Trends and Applications of Surface and Bulk Acoustic Wave Devices: A Review. Micromachines.

[21] Mujahid A., Afzal A., Dickert F.L. (2019). An Overview of High Frequency Acoustic Sensors—QCMs, SAWs and FBARs—Chemical and Biochemical Applications. Sensors.

[22] Šetka M., Bahos F.A., Matatagui D., Gràcia I., Figueras E., Drbohlavová J., Vallejos S. (2020). Love Wave Sensors with Silver Modified Polypyrrole Nanoparticles for VOCs Monitoring. Sensors.

[23] Hallil H., Omar Aouled N., Plano B., Delépée R., Agrofoglio L., Dejous C., Rebière D. (2020). Love Wave Sensor Based on Thin Film Molecularly Imprinted Polymer: Study of VOCs Adsorption. J. Integr. Circuits Syst..

[24] Ghayour R., Hojjat Y., Karafi M.R., Sadeghiyan H. (2018). Development of a Hybrid DEP-SAW Device for Trapping/Sensing Target Cells. Appl. Acoust..

[25] Chang K., Pi Y., Lu W., Wang F., Pan F., Li F., Jia S., Shi J., Deng S., Chen M. (2014). Label-Free and High-Sensitive Detection of Human Breast Cancer Cells by Aptamer-Based Leaky Surface Acoustic Wave Biosensor Array. Biosens. Bioelectron..

[26] Gagliardi M., Agostini M., Lunardelli F., Miranda A., Luminare A.G., Cervelli F., Gambineri F., Cecchini M. (2022). A Surface Acoustic Wave (SAW)-Based Lab-on-Chip for the Detection of Active α-Glycosidase. Biosensors.

[27] Han S.B., Lee S.S. (2024). Simultaneous Detection of Exosomal microRNAs Isolated from Cancer Cells Using Surface Acoustic Wave Sensor Array with High Sensitivity and Reproducibility. Micromachines.

[28] Gronewold T.M.A., Baumgartner A., Quandt E., Famulok M. (2006). Discrimination of Single Mutations in Cancer-Related Gene Fragments with a Surface Acoustic Wave Sensor. Anal. Chem..

[29] Huang Y., Das P.K., Bhethanabotla V.R. (2021). Surface Acoustic Waves in Biosensing Applications. Sens. Actuators Rep..

[30] Zhao C., Li C., Li M., Qian L., Wang L., Li H. (2022). Surface Acoustic Wave Immunosensor Based on Au-Nanoparticles-Decorated Graphene Fluidic Channel for CA125 Detection. Sens. Actuators B Chem..

[31] Lo X.-C., Li J.-Y., Lee M.-T., Yao D.-J. (2020). Frequency Shift of a SH-SAW Biosensor with Glutaraldehyde and 3-Aminopropyltriethoxysilane Functionalized Films for Detection of Epidermal Growth Factor. Biosensors.

[32] Onen O., Sisman A., Gallant N.D., Kruk P., Guldiken R. (2012). A Urinary Bcl-2 Surface Acoustic Wave Biosensor for Early Ovarian Cancer Detection. Sensors.

[33] Cheng C.-H., Yatsuda H., Goto M., Kondoh J., Liu S.-H., Wang R. (2023). Application of Shear Horizontal Surface Acoustic Wave (SH-SAW) Immunosensor in Point-of-Care Diagnosis. Biosensors.

[34] Rauf S., Qazi H.I.A., Luo J., Fu C., Tao R., Rauf S., Yang L., Li H., Fu Y. (2021). Ultrasensitive Leaky Surface Acoustic Wave Immunosensor for Real-Time Detection of Alpha-Fetoprotein in Biological Fluids. Chemosensors.

[35] Hadj-Larbi F., Serhane R. (2019). Sezawa SAW Devices: Review of Numerical-Experimental Studies and Recent Applications. Sens. Actuators A Phys..

[36] Jandas P.J., Luo J., Prabakaran K., Chen F., Fu Y.Q. (2020). Highly Stable, Love-Mode Surface Acoustic Wave Biosensor Using Au Nanoparticle-MoS2-rGO Nano-Cluster Doped Polyimide Nanocomposite for the Selective Detection of Carcinoembryonic Antigen. Mater. Chem. Phys..

[37] Tang P., Wang Y., Huo J., Lin X. (2018). Love Wave Sensor for Prostate-Specific Membrane Antigen Detection Based on Hydrophilic Molecularly-Imprinted Polymer. Polymers.

[38] Zhang F., Li S., Cao K., Wang P., Su Y., Zhu X., Wan Y. (2015). A Microfluidic Love-Wave Biosensing Device for PSA Detection Based on an Aptamer Beacon Probe. Sensors.

[39] Tigli O., Bivona L., Berg P., Zaghloul M.E. (2010). Fabrication and Characterization of a Surface-Acoustic-Wave Biosensor in CMOS Technology for Cancer Biomarker Detection. IEEE Trans. Biomed. Circuits Syst..

[40] Agostini M., Greco G., Cecchini M. (2019). Full-SAW Microfluidics-Based Lab-on-a-Chip for Biosensing. IEEE Access.

[41] Jandas P.J., Luo J., Quan A., Qiu C., Cao W., Fu C., Fu Y.Q. (2020). Highly Selective and Label-Free Love-Mode Surface Acoustic Wave Biosensor for Carcinoembryonic Antigen Detection Using a Self-Assembled Monolayer Bioreceptor. Appl. Surf. Sci..

[42] Jandas P.J., Prabakaran K., Luo J., Fu C., Fu Y.Q., Holaday M.G.D. (2021). Ti_3_C_2_T_x_ MXene-Au Nanoparticles Doped Polyimide Thin Film as a Transducing Bioreceptor for Real-Time Acoustic Detection of Carcinoembryonic Antigen. Sens. Actuators A Phys..

[43] Wang X., Ji J., Yang P., Li X., Pang Y., Lu P. (2022). A Love-Mode Surface Acoustic Wave Aptasensor with Dummy Fingers Based on Monolayer MoS2/Au NPs Nanocomposites for Alpha-Fetoprotein Detection. Talanta.

[44] Li C., Zhang J., Xie H., Luo J., Fu C., Tao R., Li H., Fu Y. (2022). Highly Sensitive Love Mode Acoustic Wave Platform with SiO_2_ Wave-Guiding Layer and Gold Nanoparticles for Detection of Carcinoembryonic Antigens. Biosensors.

[45] Zou Y. (2020). Love Wave Based Portable Sensing System for On-Line Detection of Carcinoembryonic Antigen in Exhaled Breath Condensate. Biomed. Microdevices.

[46] Sisman A., Gur E., Ozturk S., Enez B., Okur B., Toker O. (2017). A Low-Cost Biomarker-Based SAW-Biosensor Design for Early Detection of Prostate Cancer. Procedia Technol..

[47] Wang T., Green R., Nair R.R., Howell M., Mohapatra S., Guldiken R., Mohapatra S.S. (2015). Surface Acoustic Waves (SAW)-Based Biosensing for Quantification of Cell Growth in 2D and 3D Cultures. Sensors.

[48] Lee D.S., Fu Y.Q., Maeng S., Du X.Y., Tan S.C., Luo J.K., Flewitt A.J., Kim S.H., Park N.M., Choi Y.J. (2007). Integrated ZnO Surface Acoustic Wave Microfluidic and Biosensor System. Proceedings of the 2007 IEEE International Electron Devices Meeting.

[49] Murillo A.E., Melo-Máximo L., García-Farrera B., Salas Martínez O., Melo-Máximo D.V., Oliva-Ramírez J., García K., Huerta L., Oseguera J. (2019). Development of AlN Thin Films for Breast Cancer Acoustic Biosensors. J. Mater. Res. Technol..

[50] Agostini M., Greco G., Cecchini M. (2018). A Rayleigh Surface Acoustic Wave (R-SAW) Resonator Biosensor Based on Positive and Negative Reflectors with Sub-Nanomolar Limit of Detection. Sens. Actuators B Chem..

[51] Zou Y., Zhang X., An C., Ran C., Ying K., Wang P. (2014). A Point-of-Care Testing System with Love-Wave Sensor and Immunogold Staining Enhancement for Early Detection of Lung Cancer. Biomed. Microdevices.

[52] Zhang X., Zou Y., An C., Ying K., Chen X., Wang P. (2015). Sensitive Detection of Carcinoembryonic Antigen in Exhaled Breath Condensate Using Surface Acoustic Wave Immunosensor. Sens. Actuators B Chem..

[53] Hao H.-C., Chang H.-Y., Wang T.-P., Yao D.-J. (2013). Detection of Cells Captured with Antigens on Shear Horizontal Surface-Acoustic-Wave Sensors. SLAS Technol..

[54] Cai H.-L., Yang Y., Chen X., Mohammad M.A., Ye T.-X., Guo C.-R., Yi L.-T., Zhou C.-J., Liu J., Ren T.-L. (2015). A Third-Order Mode High Frequency Biosensor with Atomic Resolution. Biosens. Bioelectron..

[55] Dejous C., Hallil H., Raimbault V., Lachaud J.-L., Plano B., Delépée R., Favetta P., Agrofoglio L., Rebière D. (2016). Love Acoustic Wave-Based Devices and Molecularly-Imprinted Polymers as Versatile Sensors for Electronic Nose or Tongue for Cancer Monitoring. Sensors.

[56] Lebal N., Raimbault V., Hallil H., Plano B., Lachaud J.L., Dejous C., Rebière D., Krstulja A., Delepée R., Agrofoglio L. (2014). Love Wave-Based Acoustic Components as Versatile Sensors for Electronic Nose or Tongue. Application to Cancer Monitoring. Proceedings of the IEEE SENSORS 2014.

[57] Aouled N.O., Hallil H., Plano B., Rebiere D., Dejous C., Delepee R., Agrofoglio L. (2013). Love Wave Sensor Based on Thin Film Molecularly Imprinted Polymer: MIP Layer Morphology and Nucleosides Analogs Detection. Proceedings of the 2013 IEEE SENSORS.

[58] Zhang X.-F., Zhang Z.-W., He Y.-L., Liu Y.-X., Li S., Fang J.-Y., Zhang X.-A., Peng G. (2016). Sniffing Lung Cancer Related Biomarkers Using an Oxidized Graphene SAW Sensor. Front. Phys..

[59] Wang D., Yu K., Wang Y., Hu Y., Zhao C., Wang L., Ying K., Wang P. (2012). A Hybrid Electronic Noses’ System Based on MOS-SAW Detection Units Intended for Lung Cancer Diagnosis. J. Innov. Opt. Health Sci..

[60] He S., Gao Y., Shao J., Lu Y. (2015). Application of SAW Gas Chromatography in the Early Screening of Lung Cancer. Proceedings of the 2015 Symposium on Piezoelectricity, Acoustic Waves, and Device Applications (SPAWDA).

[61] Tsai M.-C., Tsai Y.-C. (2009). Adsorption of Glucose Oxidase at Platinum-Multiwalled Carbon Nanotube-Alumina-Coated Silica Nanocomposite for Amperometric Glucose Biosensor. Sens. Actuators B Chem..

[62] Devnani H., Sharma C., Jain P., Khan A.A.P., Kulkarni R.M., Omaish Ansari M., Asiri A.M. (2024). Immobilization Techniques in the Fabrication of Nanomaterial-Based Electrodes for Biosensing. Nanomaterial-Modified Electrodes.

[63] Pawar V.S., Pawar S.D. (2023). Advancement of Current Immobilization Techniques for Development of Recent Biosensors. Proceedings of the 2023 6th International Conference on Advances in Science and Technology (ICAST).

[64] Matatagui D., Bastida A., Horrillo M.C. (2024). Novel SH-SAW Biosensors for Ultra-Fast Recognition of Growth Factors. Biosensors.

[65] Tsou P.-H., Lin Z.-L., Pan Y.-C., Yang H.-C., Chang C.-J., Liang S.-K., Wen Y.-F., Chang C.-H., Chang L.-Y., Yu K.-L. (2021). Exploring Volatile Organic Compounds in Breath for High-Accuracy Prediction of Lung Cancer. Cancers.

[66] Saalberg Y., Wolff M. (2016). VOC Breath Biomarkers in Lung Cancer. Clin. Chim. Acta.

[67] Vassilenko V., Moura P.C., Raposo M. (2023). Diagnosis of Carcinogenic Pathologies through Breath Biomarkers: Present and Future Trends. Biomedicines.

[68] Xiang L., Wu S., Hua Q., Bao C., Liu H. (2021). Volatile Organic Compounds in Human Exhaled Breath to Diagnose Gastrointestinal Cancer: A Meta-Analysis. Front. Oncol..

[69] Li G., Zhu X., Liu J., Li S., Liu X. (2023). Metal Oxide Semiconductor Gas Sensors for Lung Cancer Diagnosis. Chemosensors.

[70] Šetka M., Bahos F.A., Matatagui D., Potoček M., Kral Z., Drbohlavová J., Gràcia I., Vallejos S. (2020). Love Wave Sensors Based on Gold Nanoparticle-Modified Polypyrrole and Their Properties to Ammonia and Ethylene. Sens. Actuators B Chem..

[71] Alves Martins B.A., de Bulhões G.F., Cavalcanti I.N., Martins M.M., de Oliveira P.G., Martins A.M.A. (2019). Biomarkers in Colorectal Cancer: The Role of Translational Proteomics Research. Front. Oncol..

[72] Zhang X., Zou Y., An C., Ying K., Chen X., Wang P. (2014). A Miniaturized Immunosensor Platform for Automatic Detection of Carcinoembryonic Antigen in EBC. Sens. Actuators B Chem..

[73] Głowska-Ciemny J., Szymański M., Kuszerska A., Malewski Z., von Kaisenberg C., Kocyłowski R. (2023). The Role of Alpha-Fetoprotein (AFP) in Contemporary Oncology: The Path from a Diagnostic Biomarker to an Anticancer Drug. Int. J. Mol. Sci..

[74] Holjencin C., Jakymiw A. (2022). MicroRNAs and Their Big Therapeutic Impacts: Delivery Strategies for Cancer Intervention. Cells.

[75] Hernández J., Thompson I.M. (2004). Prostate-Specific Antigen: A Review of the Validation of the Most Commonly Used Cancer Biomarker. Cancer.

[76] Wee P., Wang Z. (2017). Epidermal Growth Factor Receptor Cell Proliferation Signaling Pathways. Cancers.

[77] Dahmardeh M., Sheybanifar S., Gharooni M., Janmaleki M., Abdolahad M. (2015). Acoustic Wave Based Biosensor to Study Electroacoustic Based Detection of Progressive (SW-48) Colon Cancer Cells from Primary (HT-29) Cells. Sens. Actuators A Phys..

[78] Hao H.-C., Yao D.-J. (2010). A Sensitive, Rapid and Specific Technique for the Detection of Antigen-Specific Cells on Shear Horizontal Surface Acoustic Wave (SH-SAW) Sensors. Proceedings of the 2010 IEEE Sensors.

[79] Yu Y., Pan H.-Y., Zheng X., Yuan F., Zhou Y.-L., Zhang X.-X. (2022). Ultrasensitive Simultaneous Detection of Multiple Rare Modified Nucleosides as Promising Biomarkers in Low-Put Breast Cancer DNA Samples for Clinical Multi-Dimensional Diagnosis. Molecules.

[80] Rapanotti M.C., Cenci T., Scioli M.G., Cugini E., Anzillotti S., Savino L., Coletta D., Di Raimondo C., Campione E., Roselli M. (2024). Circulating Tumor Cells: Origin, Role, Current Applications, and Future Perspectives for Personalized Medicine. Biomedicines.

[81] Gruhl F.J., Rapp M., Länge K. (2010). Label-Free Detection of Breast Cancer Marker HER-2/Neu with an Acoustic Biosensor. Procedia Eng..

[82] Rana L., Gupta R., Tomar M., Gupta V. (2017). ZnO/ST-Quartz SAW Resonator: An Efficient NO_2_ Gas Sensor. Sens. Actuators B Chem..

[83] Abraham M.H., Poole C.F., Poole S.K. (1999). Classification of Stationary Phases and Other Materials by Gas Chromatography. J. Chromatogr. A.

[84] Aleixandre M., Nakamoto T. (2020). Study of Room Temperature Ionic Liquids as Gas Sensing Materials in Quartz Crystal Microbalances. Sensors.

[85] Li X., Sun W., Fu W., Lv H., Zu X., Guo Y., Gibson D., Fu Y.-Q. (2023). Advances in Sensing Mechanisms and Micro/Nanostructured Sensing Layers for Surface Acoustic Wave-Based Gas Sensors. J. Mater. Chem. A.

[86] Qureshi S., Hanif M., Jeoti V., Stojanović G.M., Khan M.T. (2024). Review of Fabrication of SAW Sensors on Flexible Substrates: Challenges and Future. Results Eng..

[87] Zhou J., Guo Y., Wang Y., Ji Z., Zhang Q., Zhuo F., Luo J., Tao R., Xie J., Reboud J. (2023). Flexible and Wearable Acoustic Wave Technologies. Appl. Phys. Rev..

[88] Liu X., Chen X., Yang Z., Xia H., Zhang C., Wei X. (2023). Surface Acoustic Wave Based Microfluidic Devices for Biological Applications. Sens. Diagn..

[89] Jeng M.-J., Li Y.-C., Sharma M., Chen C.-W., Tsai C.-L., Lin Y.-H., Huang S.-F., Chang L.-B., Lai C.-S. (2021). A Surface Acoustic Wave Sensor with a Microfluidic Channel for Detecting C-Reactive Protein. Chemosensors.

